# Supplementary material for: Wine economy in Byzantine Shivta (Negev, Israel): Exploring the role of runoff agriculture and droughts through Agent-Based Modeling
Source: PLoS One. 2025 Jul 10;20(7):e0325204. doi: 10.1371/journal.pone.0325204 (PMC12244680; doi:10.1371/journal.pone.0325204)
Supplement: S1 — This section contains all supplementary materials mentioned in the manuscript (S1 to S15). Maps throughout this article were created using ArcMap® (version 10.8.2) software by Esri, incorporating the World Imagery basemap [99]. (DOCX) [file pone.0325204.s001.docx]

**Wine economy in Byzantine Shivta (Negev, Israel): Exploring the role of runoff agriculture and droughts through Agent-Based Modeling**

**Garty, B.,^1*^ Gambash, G.,^1^ Levy, S.T.^2^ and Bar-Oz, G.^1^**

^1^ School of Archaeology and Maritime Cultures, University of Haifa, Haifa 3498838, Israel

^2^ School of Learning Sciences and Leadership in Education, University of Haifa, Haifa 3498838, Israel

* Corresponding authors: B. Garty (bargarty@gmail.com); G. Gambash (ggambash@univ.haifa.ac.il); S.T. Levy (stlevy@edu.haifa.ac.il); G. Bar-Oz (guybar@research.haifa.ac.il)

**S1: AGENTS model (v1.0.0) video tutorials**

To support the methodologies described in this article, a series of instructional videos has been created. These videos visually demonstrate key functionalities and processes of the model, offering an interactive complement to the written explanations. The video tutorials are accessible on YouTube, with direct links provided below.

**Part 1**: Introduction to model user interface, covering main controls, sliders, monitors, and the central simulation world map. <https://youtu.be/fzhIniZosUg>

**Part 2**: Detailed explanation of the setup controllers, covering adjustments for initial conditions, climate scenarios, and simulation duration. <https://youtu.be/rmeFqwEObTY>

**Part 3**: Overview of the setup routine, from initializing global variables to creating farms and applying climate and hydrology data. <https://youtu.be/-qb24YyxxMo>

**Part 4**: Breakdown of the go routine, describing the sequence of daily functions, seasonal transitions, and end-of-model run procedures. <https://youtu.be/d87EIC10YcE>

**S2: AGENTS model main data sources and key publications**

The data utilized in this thesis, both within the main text and various aspects of the code development (such as using labor costs as a proxy for energy), were derived from classical Roman texts and modern expertise. Sources include works by ancient Roman scholars such as "De Agri Cultura" by Marcus Cato, "De Re Rustica" by Marcus Terentius Varro, "De Re Rustica" by Lucius Junius Moderatus Columella, "Natural History" by Pliny the Elder, and the Nessana Papyri corpus. Additionally, consultations with hydrologists and geologists, along with interactions with contemporary grapevine growers in the Negev who practice rainfed agriculture to varying degrees, provided valuable insights and information.

# Previous studies and key publications

This research has been informed and shaped by a wealth of literature. Among the many contributions, certain studies and publications stand out for their foundational impact:

- The pioneering work of Yehuda Kedar on desert agriculture, which offers insights into ancient agricultural practices in arid environments.
- The collaborative research by Michael Evenari, Naphtali Tadmor, and Leslie Shanan on the reconstruction of ancient farms in the Negev settlements of Avdat and Shivta, providing practical perspectives on ancient farming techniques and strategies.
- Richard Duncan-Jones' "The Economy of The Roman Empire: Quantitative Studies," which provides a comprehensive overview of agriculture in ancient Rome and serves as a valuable reference for economic and agricultural context.
- "An Introduction to Agent-Based Modeling" by Uri Wilensky and William Rand, which served as a foundational text for learning to code in NetLogo and initiating model development for this thesis.
- "Agent-Based Modeling for Archaeology" by Iza Romanowska, Colin Wren, and Stefani Crabtree, which was instrumental in guiding the development of the thesis model to align with archaeological context and accepted practices.

**S3: AGENTS model precipitation dataset preparation process**

**Fig. A** below illustrates correlation data between Shivta (station ID 252920) and Sede Boqer (station ID 253000) Israel Metrological Services (IMS) stations taken from the IMS archive [1].

Daily precipitation data for Shivta (station ID 252920) and Sede Boqer (station ID 253000) for the years 1963–1971 were downloaded from the Israel Meteorological Service (IMS) archive [1]. The datasets were compared, and a strong correlation was observed (R² 0.83). This relationship allowed for the derivation of a correlation equation to estimate Shivta's precipitation using Sede Boqer’s data. Refer to **Fig. A** and **Equation A** (in S3), that was derived from this correlation allowing to calculate Shivta’s missing precipitation data for the full range between 1952 to 2022.


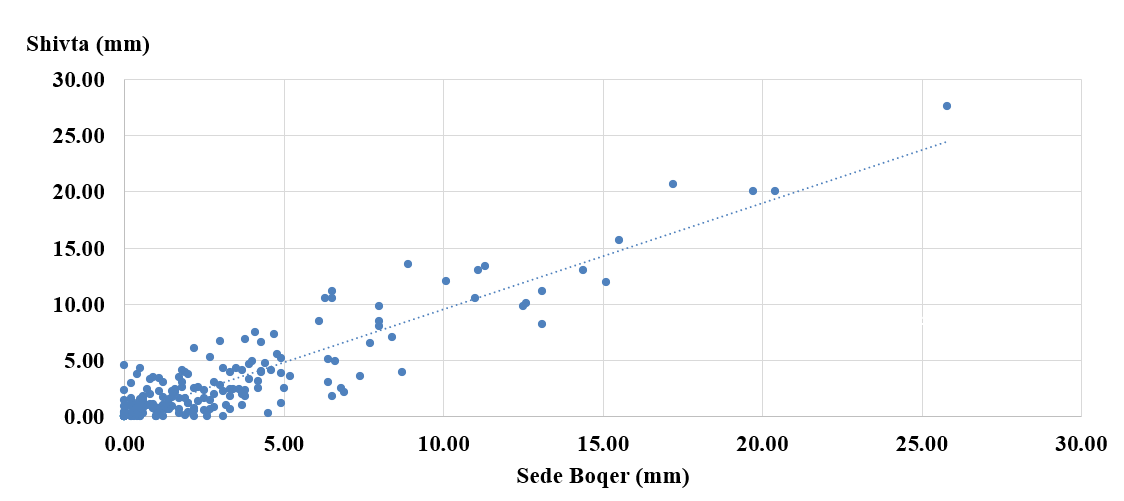


**Fig. A: Sede Boqer vs. Shivta daily rainfall (mm) correlation 1963−1971 (N = 208)**

**Equation A: Shivta daily precipitation (mm) as function of Sede Boqer daily precipitation (mm)**

| $\boldsymbol{Precipitation}_{\left( \mathbf{Shivta; mm} \right)}\boldsymbol{=0.943\times}\boldsymbol{Precipitation}_{\left( \mathbf{Sede Boqer; mm} \right)}\boldsymbol{+0.132}$ |
| --- |


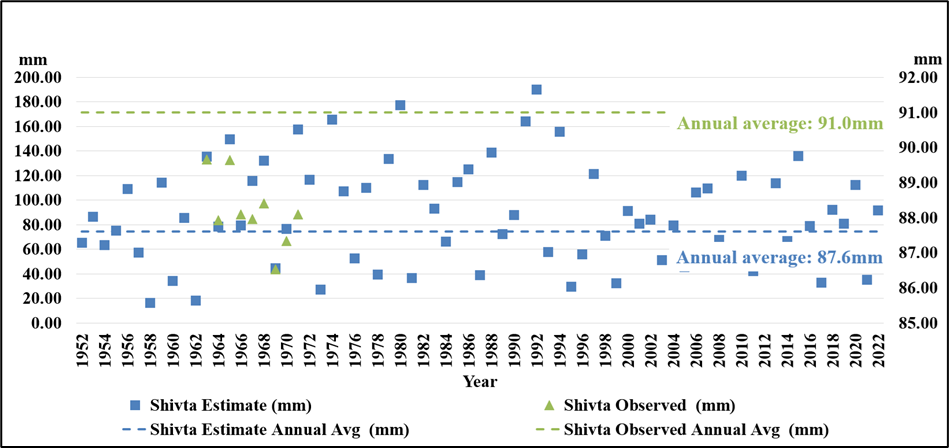


**Fig. B:** **Comparison of observed (1963–1971) and estimated (1952−2022) annual rainfall in Shivta (N = 71)**

**Table A: Shivta annual precipitation reconstructed dataset**

| **Year** | **mm** |  | **Year** | **mm** |  | **Year** | **mm** |  | **Year** | **mm** |
| --- | --- | --- | --- | --- | --- | --- | --- | --- | --- | --- |
| 1952 | 65.3 |  | 1973 | 27.4 |  | 1994 | 155.6 |  | 2015 | 136.0 |
| 1953 | 86.4 |  | 1974 | 165.5 |  | 1995 | 29.6 |  | 2016 | 78.8 |
| 1954 | 63.4 |  | 1975 | 107.3 |  | 1996 | 55.9 |  | 2017 | 32.7 |
| 1955 | 75.4 |  | 1976 | 52.8 |  | 1997 | 121.3 |  | 2018 | 92.0 |
| 1956 | 109.3 |  | 1977 | 110.3 |  | 1998 | 70.9 |  | 2019 | 80.9 |
| 1957 | 57.3 |  | 1978 | 39.5 |  | 1999 | 32.2 |  | 2020 | 112.2 |
| 1958 | 16.6 |  | 1979 | 133.4 |  | 2000 | 91.2 |  | 2021 | 35.4 |
| 1959 | 114.2 |  | 1980 | 177.2 |  | 2001 | 80.7 |  | 2022 | 91.5 |
| 1960 | 34.2 |  | 1981 | 36.6 |  | 2002 | 84.4 |  |  |  |
| 1961 | 85.5 |  | 1982 | 112.5 |  | 2003 | 51.1 |  |  |  |
| 1962 | 18.4 |  | 1983 | 93.2 |  | 2004 | 79.6 |  |  |  |
| 1963 | 135.6 |  | 1984 | 66.2 |  | 2005 | 45.7 |  |  |  |
| 1964 | 77.6 |  | 1985 | 114.9 |  | 2006 | 106.3 |  |  |  |
| 1965 | 149.7 |  | 1986 | 125.0 |  | 2007 | 109.4 |  |  |  |
| 1966 | 79.6 |  | 1987 | 38.9 |  | 2008 | 67.5 |  |  |  |
| 1967 | 115.8 |  | 1988 | 137.9 |  | 2009 | 52.3 |  |  |  |
| 1968 | 131.9 |  | 1989 | 72.2 |  | 2010 | 120.0 |  |  |  |
| 1969 | 44.7 |  | 1990 | 87.9 |  | 2011 | 42.1 |  |  |  |
| 1970 | 76.5 |  | 1991 | 164.0 |  | 2012 | 61.2 |  |  |  |
| 1971 | 157.4 |  | 1992 | 189.4 |  | 2013 | 113.9 |  |  |  |
| 1972 | 116.8 |  | 1993 | 57.9 |  | 2014 | 66.8 |  |  |  |

**S4: AGENTS model evaporation dataset preparation process**

**Fig. C** illustrates the strong correlation (R² = 0.98) between daily mean temperatures recorded at the Israel Meteorological Services (IMS) stations in Shivta (ID 252920) and Beer Sheva (ID 7840). Data for both stations were downloaded from the IMS archive [1], and daily mean temperature (°C) was calculated as the average of the recorded minimum and maximum temperatures for each day.


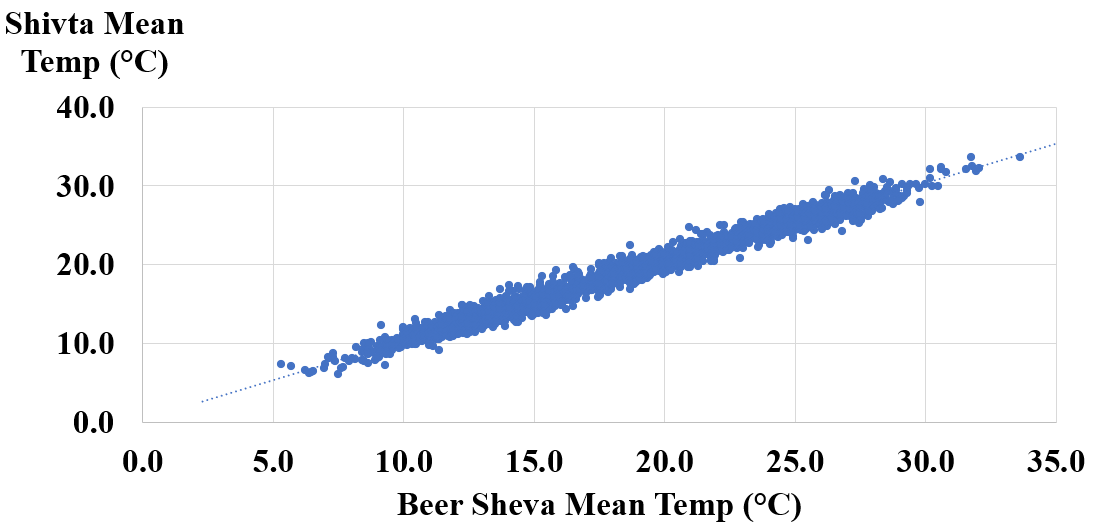


**Fig. C: Shivta vs. Beer Sheva daily mean temperature (°C; 1965−1971; N=2,462)**

**Fig. D** shows a moderate correlation (R² = 0.73) between daily mean temperature (°C) and daily evaporation (mm) recorded at the Be’er Sheva IMS station (ID 7840) for the years 1964–1999. To improve the fit, data points with evaporation record codes of 1 or 2 (non-actual measurements) were excluded. This correlation enabled the derivation of a linear equation (refer to **Equation B** below) used to estimate daily evaporation (mm) for the years 1952–2022 based on temperature data from IMS stations 7840, 7841, 251692, and 251850 [1]. This dataset was exported as a CSV file and incorporated into the AGENTS model for Shivta’s Zetan watershed daily evaporation simulations.


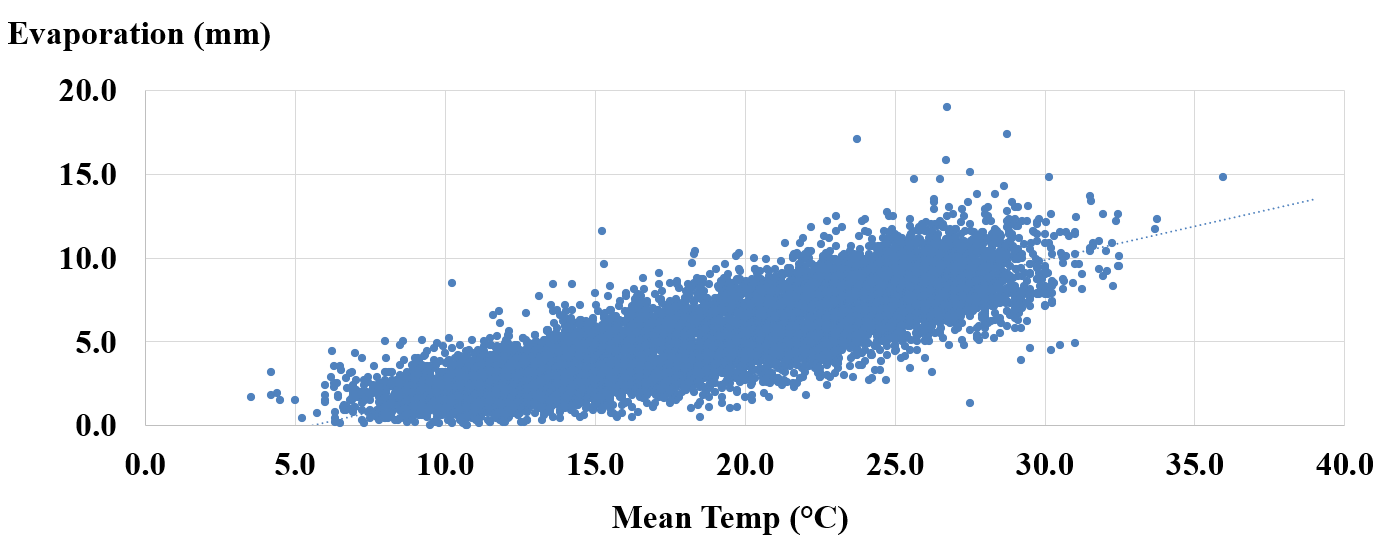


**Fig. D: Beer Sheva daily evaporation (mm) as function of mean daily temperature (°C) for 1964−1999 (N=10,679)**

**Equation B: Beer sheva daily evaporation (mm) as function of daily mean temperature (°C)**

| $\boldsymbol{Evaporation}_{\boldsymbol{daily(mm);Beer Sheva}}\boldsymbol{=0.4032*}\boldsymbol{T}_{\boldsymbol{daily mean}\boldsymbol{(^{\circ}C}\boldsymbol{);Beer Sheva}}\boldsymbol{-2.2316}$ |
| --- |

**S5: AGENTS model curve number method data**

**(1) Curve Number Method terminology**

**S**: Maximum potential retention after runoff begins (mm), related to soil and land cover properties.

**P**: Total precipitation (mm) for a rainfall event.

**Q**: Actual runoff depth (mm) resulting from a rainfall event.

**Ia**: Initial abstraction (mm), including water losses before runoff, such as infiltration and surface storage.

**λ**: Ratio of initial abstraction (Ia) to maximum potential retention (S), typically assumed as 0.2.

**CN**: Curve Number for different soil moisture conditions; 1 (dry), 2 (average), 3 (wet).

**Antecedent Moisture Condition (AMC)**: Classification of soil moisture prior to a rainfall event; AMC 1 (dry conditions), AMC 2 (average conditions), AMC 3 (wet conditions).

The AGENTS model uses λ = 0.05, which reduces error margins when comparing rainfall (P) to runoff (Q) against observed datasets [2−3]. This value is more suitable for small watersheds that require lower CN values or have land cover types, such as forested or desert soils, that are underrepresented in the original SCS land use–land cover curve number index tables [4].

For runoff calculations, the AGENTS model, designed to simulate the climate and runoff in Shivta's Zetan watershed desert topography, considers only AMC 1 (dry conditions) and AMC 2 (average conditions).

**(2) AGENTS Model Curve Number Equations**

**Equation C:** **Wet soil conditions (CN_3_) curve number index [5]**

| $\boldsymbol{CN}_{\boldsymbol{3}}\boldsymbol{=}\frac{\boldsymbol{CN}_{\boldsymbol{2}}}{\boldsymbol{0.427+0.00573*}\boldsymbol{CN}_{\boldsymbol{2}}}$ |
| --- |

Refer to Table 1 in the Methods section of the main article for CN_2_ values.

**Equation D: Slope-adjusted curve number index for normal soil moisture conditions (CN_2_; [6])**

| $\boldsymbol{CN}_{\boldsymbol{2-slope}}\boldsymbol{=}\left( \boldsymbol{0.5-0.714*}\boldsymbol{exp}^{\boldsymbol{-7.125*slope}} \right)\boldsymbol{*}\left( \boldsymbol{CN}_{\boldsymbol{3}}\boldsymbol{-}\boldsymbol{CN}_{\boldsymbol{2}} \right)\boldsymbol{+}\boldsymbol{CN}_{\boldsymbol{2}}$ |
| --- |

Slope is measured as percentage-rise values.

**Equation E: Slope-adjusted curve number index for dry soil moisture conditions (CN_1_;** **[5])**

| $\boldsymbol{CN}_{\boldsymbol{1-slope}}\boldsymbol{=}\frac{\boldsymbol{CN}_{\boldsymbol{2-slope}}}{\boldsymbol{2.281-0.01281*}\boldsymbol{CN}_{\boldsymbol{2-slope}}}$ |
| --- |

**Equation F: Soil maximum potential retention (S, mm) as function of soil normal moisture conditions curve number index (CN; [7])**

| $\boldsymbol{S=}\frac{\boldsymbol{25,400}}{\boldsymbol{CN}}\boldsymbol{-254}$ |
| --- |

S is calculated using CN values specific to soil moisture conditions: CN1-slope (**Equation E**) for AMC1 (dry conditions) and CN2-slope (**Equation D**) for AMC2 (normal conditions).

**Equation G: Soil maximum potential retention (S, mm) conversion from λ=0.20 to λ=0.05 [8]**

| $\boldsymbol{S}_{\boldsymbol{\lambda=0.05}}\boldsymbol{=1.42*}\boldsymbol{S}_{\boldsymbol{\lambda=0.20}}$ |
| --- |

**Equation H: Initial abstraction (I_a_, mm) as function of λ and Soil maximum potential retention (S, mm) [7].**

| $\boldsymbol{I}_{\boldsymbol{a}}\boldsymbol{=}\boldsymbol{\lambda}\boldsymbol{*}\boldsymbol{S}_{\boldsymbol{\lambda=0.05}}$ |
| --- |

Where λ = 0.05 and S_λ=0.05_​ is calculated using **Equation G**. For AMC_1_ (dry conditions), Ia uses S calculated with CN1-slope (**Equation E**) for AMC1 (dry conditions) and CN2-slope (**Equation D**) for AMC2 (normal conditions), Ia uses S calculated with CN_2-slope_ (**Equation D**).

**Equation I: Runoff amount (Q, mm) calculation as function of rainfall (P, mm) and Soil maximum potential retention (S, mm) [7].**

| $\boldsymbol{Q=}\left\{ \begin{matrix} \frac{\boldsymbol{(P-0.05*S)}^{\boldsymbol{2}}}{\boldsymbol{P+0.95*S}} & \boldsymbol{P>0.05*S} \\ \boldsymbol{0} & \boldsymbol{otherwise} \end{matrix} \right.$ |
| --- |

**(3)** **AGENTS Model Patch Antecedent Moisture Condition (AMC) Values**

Antecedent moisture condition (AMC) represents soil saturation levels prior to a storm. Saturated soil contributes more rainfall to runoff, while drier soil absorbs more, reducing runoff potential. In the AGENTS model, AMC is determined based on the cumulative precipitation over the previous 5 days, following the approach outlined by Mishra & Singh [9].

Each patch in the AGENTS model calculates its 5-day soil moisture level by summing the daily infiltration, which is the rainfall amount (mm) minus the runoff (mm) and daily evaporation (mm). If this cumulative value exceeds the patch's initial abstraction (Ia) for dry conditions, the patch is assigned AMC2, indicating wetter soil. Otherwise, it is assigned AMC1, indicating dry soil conditions.

Runoff (Q, mm) for each patch is then calculated based on its current AMC value (AMC1 or AMC2) using **Equation I**. This calculation is updated daily during each tick of the simulation, where one tick represents a single day in the AGENTS model timeline.

**S6: AGENTS model runoff amounts calibration based on curve number indices**

**Fig. E** below shows the Zetan study area within the larger Nessana watershed, along with the locations of the hydrometric and meteorological stations used for calibration.


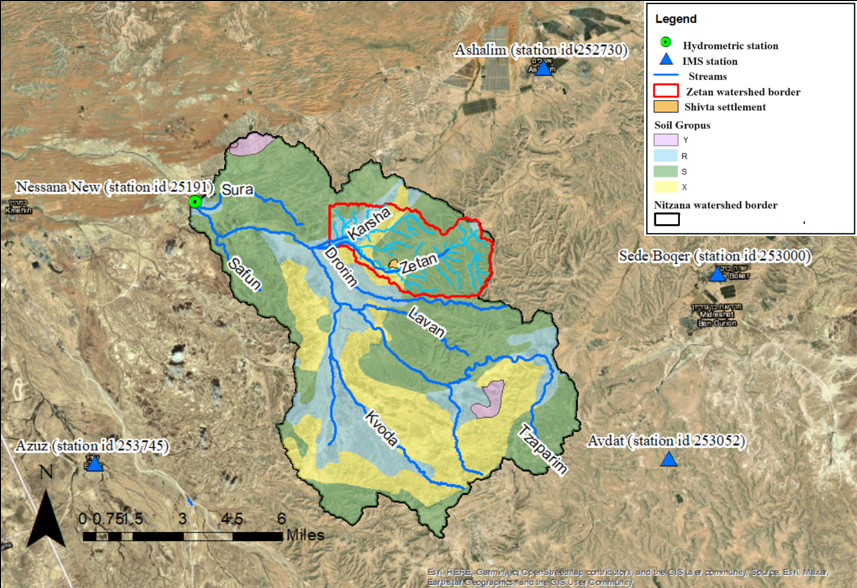


**Fig. E: Nessana hydrometric station watershed, Zetan watershed and IMS stations**

**Basemap source: Esri, Maxar, Earthstar Geographics, and the GIS User Community.**

The Zetan basin, outlined in red, spans 25.08 km² and shares similar soil group ratios with the Nessana watershed, which covers 207 km². The predominant soil type in both watersheds is Brown Lithosols (hydrologic group S), with the remainder evenly split between Loessial Serozems (group R) and Bare Rocks/Desert Lithosols (group X). Detailed soil group distributions are listed in **Table B**.

**Table B: Soil Group Distribution (%) and Weighted CN Index for Nessana and Zetan Watersheds [10]**

| **Soil Group (CN2 index)** | **Nessana Watershed** | **Zetan Watershed** |
| --- | --- | --- |
| **R (77)** | 21% | 14% |
| **S (85)** | 52% | 72% |
| **X (92)** | 25% | 14% |
| **Others** | 1% | 0% |
| **Weighted CN index** | 83.4 | 84.9 |
| **Area (km²)** | 207 | 25.08 |

**Fig. F** illustrates a strong quadratic correlation (R² = 0.84) between the average rainfall depth (mm) from four surrounding IMS stations [1] and the runoff recorded at the Nessana hydrometric station [11], scaled to the Zetan watershed's contribution (12% of total runoff).


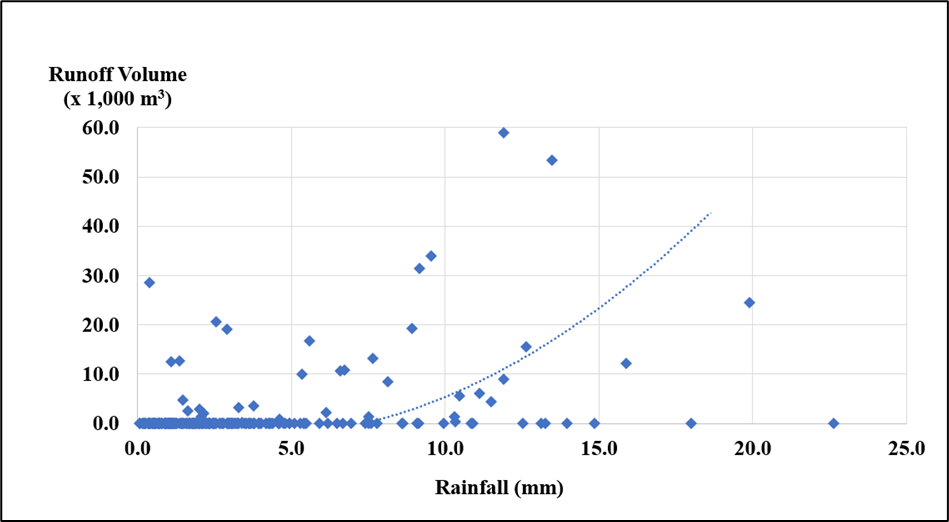


**Fig. F: Shivta area average precipitation (mm) vs. Zetan watershed runoff volume (2009−2023; N=239)**

From this correlation, **Equation J** was derived, indicating that a minimum rainfall depth (initial abstraction) of 7.34 mm is required for runoff to occur.

**Equation J: runoff volume (unit^3^) as function of total rainfall (unit)**

| $\boldsymbol{runoff\_volume=203.91*}\boldsymbol{rainfall}^{\boldsymbol{2}}\boldsymbol{-1498.5*rainfall+7.3429}$ |
| --- |

All rainfall events with daily depths exceeding 7.34 mm (N = 225) from the AGENTS model precipitation dataset (1952–2022) were used to calculate runoff volumes using **Equation J**. The same data was simulated in the AGENTS model, and the generated runoff volumes for each event were recorded. The two datasets showed a high correlation (R² = 0.99), validating the model's output. Refer to **Fig. G**.


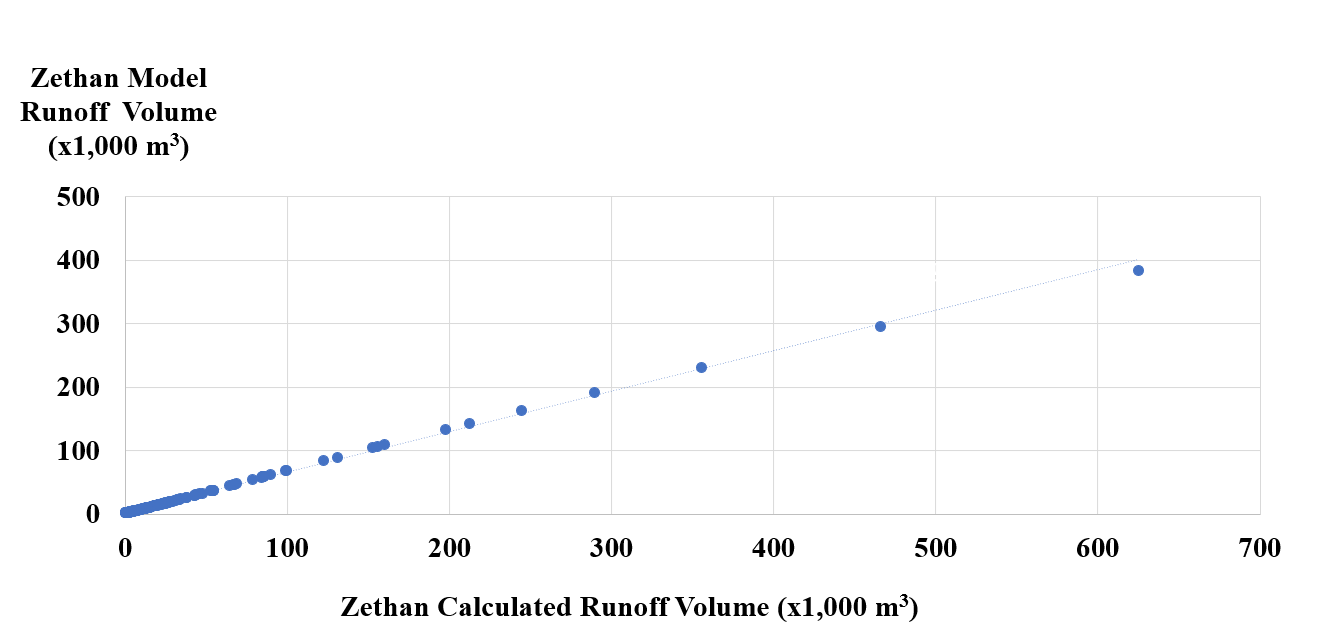


**Fig. G: Modeled vs. observed Zetan watershed runoff volume (1952−2022; N=225)**

The AGENTS model runoff volume was found to be ~63.8% of the real-world runoff volume. To account for this discrepancy, a correction factor of 1.567 was introduced, multiplying all generated runoff amounts (in AGENTS model simulations). **Fig. H** compares AGENTS model runoff volumes before and after applying this multiplier with observed runoff volumes.


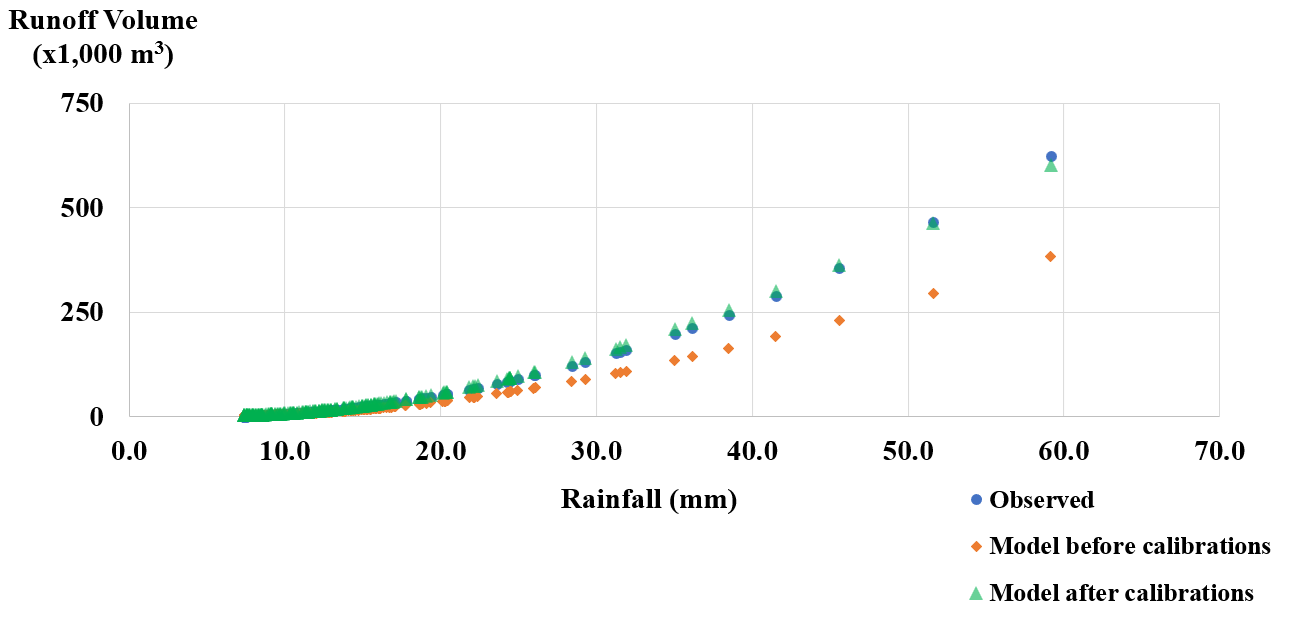


**Fig. H:** **Observed and modeled Zetan runoff volume (m^3^) as function of Shivta rainfall depth (mm) (1952-2022; N=225)**

**S7: AGENTS model crop yield equations for wheat and grapevines**

**(1) Grapevine**

Rainfed grapevine yield data for Castilla-La Mancha (central Spain) was obtained from the regional farmers' union website [12] for the years 2010–2018. This data was correlated with annual rainfall reported for the same region and time period [13], showing a strong quadratic fit (R² = 0.74). Refer to **Fig. I**.


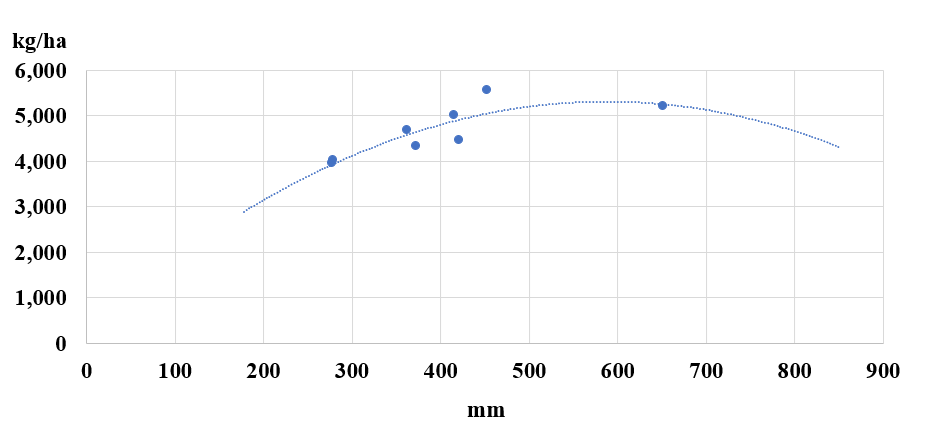


**Fig. I: Annual yield of rainfed grapevines (kg/ha) vs. annual precipitation (mm) for Castilla-La Mancha (Spain), 2010–2018**

From this correlation (**Fig. I**), a quadratic equation was derived to calculate grapevine yield as a function of **Terrace Available Water Storage (T_AWS_)**. T_AWS_ represents the amount of infiltrated water accumulated in a terrace over a single AGENTS model season. To adapt the equation to the AGENTS model, the yield values were scaled by a factor of 0.09 to convert data from kilograms per hectare to the terrace area used in the model. Refer to **Equation K**.

**Equation K: AGENTS model grapes yield as function of Terrace Available Water Storage (T_AWS_)**

| $\boldsymbol{yield}_{\boldsymbol{grapes}}\boldsymbol{=}\left[ \boldsymbol{-0.0144\times}\left( \boldsymbol{T}_{\boldsymbol{AWS}} \right)^{\boldsymbol{2}}\boldsymbol{+16.931\times}\left( \boldsymbol{T}_{\boldsymbol{AWS}} \right)\boldsymbol{+340.68} \right]\boldsymbol{\times0.09}$ |
| --- |

In the AGENTS model, thresholds were introduced to nullify yields when T_AWS_ values fall below 200 mm or exceed 1,000 mm per season, following the guidelines of Jackson & Schuster [14] and further supported by Kedar [15]. These thresholds account for the biological limits of grapevine productivity, where insufficient water leads to crop failure and excessive water impairs growth and yield.

**(2) Wheat**

**Fig. J** shows the relationship between rainfed wheat yield (kg/ha) and annual precipitation (mm), based on studies conducted in the Negev and other semi-arid regions of Israel [16].


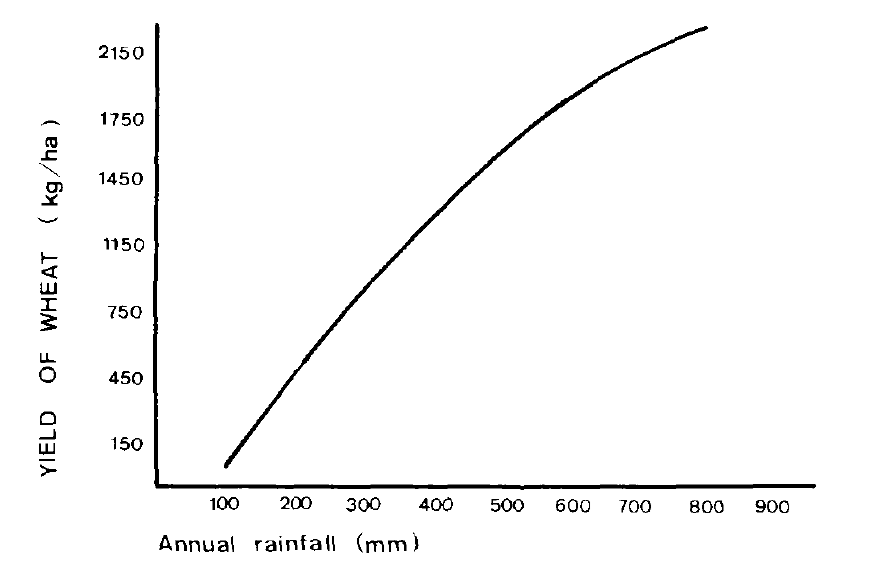


**Fig. J: Rainfed wheat yield (kg/ha) as a function of annual rainfall (mm) [16]**

Using PlotDigitizer [17], a quadratic equation (**Equation L**) was derived from the data (**Fig. J** curve) and integrated into the AGENTS model. The equation was implemented in the same manner as the grapevine yield equation, linking wheat yield to available water storage. To account for the practice of growing wheat in alternate years (fallowing), the AGENTS model multiplies each wheat field's seasonal yield by 0.5, simulating a scenario where half the field is sown while the other half is left fallow to rest for one year.

**Equation L: AGENTS model wheat yield as function of Terrace Available Water Storage (T_AWS_)**

| $\boldsymbol{yield}_{\boldsymbol{wheat}}\boldsymbol{=}\left[ \boldsymbol{-0.0023\times}\left( \boldsymbol{T}_{\boldsymbol{AWS}} \right)^{\boldsymbol{2}}\boldsymbol{+5.1382\times}\left( \boldsymbol{T}_{\boldsymbol{AWS}} \right)\boldsymbol{-414.47} \right]\boldsymbol{\times0.09\times0.5}$ |
| --- |

Additionally, to reflect the absence of pest control available to Byzantine Shivta farmers, a further 10% yield reduction was applied to both crops. See **Table C** below for example of this scaling of maximum grape yield per unit area.

**Table C: Maximum yield adjustments for grapevine yield efficiency calculations**

| Value | Adjustment |
| --- | --- |
| T_AWS_ (max) = 588mm | First-order derivative of the yield formula, representing the maximum Total Annual Water Supply (TAWS). |
| Yield _TAWS (max)_ = 5,317 kg/ha | Calculate maximum yield per hectare using the yield formula with the TAWS (max) value. |
| Yield _TAWS (max)_ = 479 kg/terrace | Convert the yield to the AGENTS model terrace area (900 m²; DEM patch resolution size) by multiplying by 0.09. |
| Yield _TAWS (max)_ = **432 kg/terrace** | Adjust for the absence of pest control in the Byzantine period by applying a 10% reduction (multiply by 0.9). |

**S8: AGENTS Model Labor Cost Calculations for Grapevine and Wheat Farming Tasks**

Creating Wheat Fields

Based on Evenari’s descriptions of ancient farm reconstructions [18:179–190], we estimated that wheat fields were typically enclosed by low earthen walls, approximately 20–30 cm high and twice as wide, forming a mound- or half-circle-shaped border around the field.

In the AGENTS model, a standard field (patch) measures 30 m × 30 m. To enclose such a field, approximately 120 meters of earthen wall would be needed. Assuming a half-circular mound shape with a cross-sectional radius of 0.25 m, the soil volume required for the enclosure is calculated as:

$$\Pi*\left( 0.25m \right)^{2}* 0.5* 120m=11.8m^{3}$$

According to Erasmus [19:284–285], a worker can excavate approximately 5 cubic meters of soil per day. Therefore, the labor required to construct such an enclosure for a 0.1-hectare wheat field (adjusted from a single patch of 0.09 ha) is calculated as follows:

$$\frac{11.8\frac{m^{3}}{patch}}{5\frac{m^{3}}{man-days}}*\frac{0.1ha}{0.09\frac{ha}{patch}}\approx2.6man-days$$

To account for the additional labor needed to construct runoff diversion features—such as feeder and sub-channels—as described by Evenari [18:179–190], we added approximately 50% (1.4 man-days) to the estimate.

Thus, the total labor required to prepare a 0.1 ha wheat field was estimated at 4 man-days.

Clearing a runoff field

Based on Evenari’s estimate [18:132], clearing a 1-hectare runoff field required between 200 and 300 man-days of labor. Accordingly, clearing a 0.1-hectare field would require 20–30 man-days. The AGENTS model uses the midpoint of this range—25 man-days per 0.1 ha—equivalent to 22.5 kg of wheat in labor cost per runoff plot, as defined by the model’s runoff ratio setting. For example, if the runoff ratio is set to 1:20, the cost of clearing runoff area for each vineyard plot would be 20 times the base cost of 22.5 kg of wheat, totalling 450 kg.

Wheat field season agriculture activities

Columella (*Rust*. 2.12.1) lists several tasks required annually for maintaining one *iugerum* of wheat, totaling 10.5 man-days of labor.

Duncan-Jones [20: 329] adds 2–3 man-days per *iugerum* for threshing—a crucial process for separating wheat grains from the chaff—and an additional 1 man-day for cutting straw. This results in a combined total of 3.5 additional man-days.

Spurr [21: 138] and Rosenstein [22: 68] argue that 4–6 more man-days should be added for tasks not explicitly mentioned by Columella, such as manuring, transporting the grain to the threshing floor, gathering hay, and similar duties.

Altogether, these sources suggest a total labor requirement of approximately 20 man-days per *iugerum*, which translates to 8 man-days per 0.1 hectare (noting that 1 *iugerum* ≈ 0.25 hectares).

For wheat farming, the AGENTS model applies a reduced cost in kg of wheat, since fields are managed with a fallow system. Each patch alternates annually between cultivation and rest, meaning only half a patch is active in any given season. Therefore, the cost per 0.1 ha is halved, amounting to 3.6 kg of wheat.

Wheat sown

According to Varro (*Rust*. 1.44.1), wheat was typically sown at a rate of 5 *modii* per *iugerum*. Pliny the Elder (*HN* 18.12) provides a range of wheat varieties from different regions with varying weights per *modius*. We adopted the average value from his accounts—22.5 pounds per *modius*. Using a conversion of 328.9 grams per pound, the total seed weight per *iugerum* can be calculated. To express this in terms of 0.1 hectares (noting that 1 *iugerum* ≈ 0.25 hectares), the equation is as follows:

$$\frac{5\frac{modii}{iugerum}*22.5\frac{pound}{modius}*328.9\frac{gram}{pound}}{2.5\frac{0.1ha}{iugerum}*1,000\frac{gram}{kg}})=14.8\frac{kg wheat}{0.1ha}$$

Thus, sowing one 0.1 ha wheat field would require approximately 14.8 kg of wheat; however, under the fallow system used in the AGENTS model, this amounts to 7.4 kg per 0.1 ha.

Vineyard: soil preparation, digging holes and planting vines

According to Duncan-Jones [20: 331] accounting for Columella work from both *De Re Rustica* and *De Arboribus*, the number of vine holes that can be dug in one man-day per *iugerum* depends on hole size: 12 or14 holes of 4 square feet, 18 holes of 3 square feet, or 20 holes of 2 square feet. This provides a range of 12 to 20 holes per man-day per *iugerum*. Converting this to a 0.1 ha area (noting that 1 *iugerum* ≈ 0.25 ha) yields:

$$Min: 12\frac{holes}{man-day*iugerum}*0.4\frac{iugerum}{0.1ha}=4.8\frac{holes}{man-day*0.1ha}$$

$$Max: 20\frac{holes}{man-day*iugerum}*0.4\frac{iugerum}{0.1ha}=8.0\frac{holes}{man-day*0.1ha}$$

Planting density in the model is based on modern vineyard standards to align with the yield equation, which is also drawn from modern rainfed viticulture. Standard spacing is 3 meters between rows and 1.5 meters between vines within rows, resulting in 2,220 vines per hectare, or 222 vines per 0.1 hectare.

Therefore, digging 222 holes for planting in a 0.1 ha vineyard plot would require:

$$Min: \frac{222\frac{holes}{0.1ha}}{8.0\frac{holes}{man-day*0.1ha}}=27.8 man-days$$

$$Max: \frac{222\frac{holes}{0.1ha}}{4.8\frac{holes}{man-day*0.1ha}}=46.3 man-days$$

In the AGENTS model, we adopted the value of 38 man-days for digging 222 holes, approximately the mid-point of that min-max range, or 34.2kg of wheat in labor costs per 0.1ha.

Vineyard: age 1–3 maintenance (excluding harvest)

Duncan-Jones [20: 331], accounting for Columella work from both *De Re Rustica* and *De Arboribus*, lists the annual agriculture activities required for a vineyard—such as soil loosening, hoeing, and pruning—at 23.5 man-days per *iugerum*. Spur [21: 136] adds an additional 12 man-days per *iugerum* to that estimate to account for harvest labor (7 man-days) and other minor tasks. Duncan-Jones [20: 331], citing other primary sources (Cato, Pliny, Saserna and Varro), suggests that total annual upkeep, including harvest, could reach a maximum of 44.5 man-days per *iugerum*.

To isolate only the maintenance (excluding harvest), the labor estimates per *iugerum* are:

**Minimum**: 23.5 + (12 − 7) = 28.5 man-days, or 11.4 man-days per 0.1 ha

**Maximum**: 44.5 − 7 = 37.5 man-days, or 15.0 man-days per 0.1 ha

The AGENTS model utilizes the midpoint of this range—13.2 man-days per 0.1 ha—as the annual agriculture activities labor cost. To account for the first three years of vine growth, this figure is multiplied by three, resulting in a total of 39.6 man-days. In the AGENTS model code, this labor input is represented as 35.6 kg of wheat equivalent.

Vineyard: age 4+ maintenance (including harvest)

By adding the harvest labor cost of 7 man-days per *iugerum* (equivalent to 2.8 man-days per 0.1 ha), the total labor required for the annual agriculture activities of a vineyard ranges between 14.2 and 17.8 man-days per 0.1 ha. In the AGENTS model, we used the midpoint of this range—16 man-days—which corresponds to 14.4 kg of wheat. This value was applied in the model to represent the annual agriculture activities cost of vineyards aged four years or older.

**S9: Glossary**

**Agent-Based Model (ABM)** – A computational model for simulating the actions and interactions of autonomous agents to assess their effects on the system as a whole.

**Annual Precipitation** – The total amount of rainfall received in a year, often expressed in millimeters (mm).

**ArcGIS Map** – A geospatial mapping software developed by Esri that allows users to visualize, analyze, and manage geographic data. It is widely used for creating, editing, and sharing spatial data and maps in various disciplines, including environmental science, archaeology, and urban planning.

**Average yield (kg/terrace)** – The average production per active terrace for a given crop type (grapevine or wheat) on a farm at the end of a season. This is calculated by dividing the farm’s total yield for the crop type by the number of active terraces producing that crop. The average yield is then compared against predefined failure and success thresholds, which are used to update the farm’s historical record of yield performance for each crop type. These updates influence the farm’s decision-making process regarding future crop expansion.

**BehaviorSpace** – A NetLogo application that allows users to run experiments with their models by varying parameters systematically.

**Consecutive Droughts** – Extended periods of below-average precipitation occurring without interruption, which can significantly impact agricultural productivity.

**Curve Number (CN, dimensionless)** – A numerical value used in hydrology to predict direct runoff or infiltration from rainfall, based on land use, soil type, and hydrologic condition.

**Digital Elevation Model (DEM)** – A 3D representation of a terrain's surface created from terrain elevation data.

**Drought Threshold** – The specific level of annual precipitation below which conditions are classified as a drought. In this study, a drought is defined as receiving less than 66 mm of annual rainfall, based on regional climatic studies [23].

**Evapotranspiration** – The combined process of evaporation from soil and water surfaces and transpiration from plants.

**Geographical Information Systems (GIS)** – Systems designed to capture, store, manipulate, analyze, manage, and present spatial or geographic data.

**Honestly Significant Difference (HSD)** – A statistical test to determine if the means of different groups are significantly different from each other.

**Hydrological Soil Group** **(HSG)** – A classification of soils based on their infiltration and runoff potential, influencing water movement.

**Infiltration rate (mm/hour)** – The rate at which water enters the soil.

**Initial abstraction (Ia, mm)** – Initial amount of precipitation before runoff begins.

**Initial abstraction ratio (λ, dimensionless)** – The ratio of initial abstraction to total rainfall.

**Land Use Land Cover (LULC)** – The categorization of land based on its use and cover type.

**Loss Aversion** – A behavioral economics concept where losses are perceived as more impactful than equivalent gains.

**Man-Days** – A unit of measurement representing the amount of work one person can complete in a day.

**NetLogo** – A programming environment used for developing agent-based models, particularly suited for simulating complex systems.

**Patch** – Special kind of stationary agents in NetLogo that make up the world of a model.

**Potential maximum retention (S, mm)** – Also infiltration potential, it is the maximum amount of water that soil can retain before runoff occurs.

**Prospect Theory** – A theory from behavioral economics that describes how people choose between probabilistic alternatives involving risk.

**Rainfall depth** **(mm)** – The total amount of rainfall accumulated over a specific area and time period.

**Run period** **(years)** – The model simulation set time range.

**Runoff depth** **(mm)** – The total amount of water from rainfall that flows over the land surface.

**Runoff farm** – A catchment basin and terraced fields together constitute an indivisible entity: the runoff-farm unit.

**Runoff ratio** **(dimensionless)** – The ratio between producing areas (hillslopes surrounding a lower wadi area) that channel water to a lower receiving area, such as an agricultural terrace located in a wadi.

**Recovery Time (years)** – The time required for an agricultural system or industry to return to pre-drought production levels after the cessation of a drought.

**Soil Conservation Service Curve Number (SCS-CN) Method** – A hydrological model used to estimate runoff based on land use, soil type, and hydrological conditions.

**SPSS Analysis** – Statistical analysis conducted using SPSS (Statistical Package for the Social Sciences), a software tool for data analysis.

**Stone-mound (Tullilat – el – Anab)** – Piles of stones created as a by-product of clearing rocks from slopes to enhance runoff collection. This practice was part of a broader ancient agricultural system, where the cleared areas served as runoff harvesting zones, directing water to cultivated areas in nearby streambeds.

**Surface runoff (Q, mm)** – The water flow that occurs when soil is saturated and excess water flows over the land surface.

**Tick** **(time unit)** – A measure of time in NetLogo models.

**Yield** – The amount of crop produced per unit area of land (i.e., kg per dunam).

**Total Yield (kg)** – The total production of each crop type (grapevine or wheat) for a farm at the end of a season. This value is calculated by summing the yields from all active terraces owned by the farm for a specific crop type. The result is recorded for each season, representing the farm’s overall production performance.

**Yield efficiency** **(fractional form between 0 and 1)** – The ratio representing the actual crop yield of a cultivated terrace compared to the maximum potential yield of that crop under optimal conditions. This terrace-specific measure is calculated by dividing the actual yield achieved during a season by the maximum theoretical yield for the crop type (430.7 kg for grapevines, 103.5 kg for wheat). The resulting proportion reflects the terrace's performance as a percentage of its maximum potential, offering insight into the effectiveness of agricultural practices and environmental conditions.

**S10:** **AGENTS model crop yield evaluation for wheat and grapevines**

Both grapevine yield formula and wheat yield formula adopted in the AGENTS model were thoroughly assessed. The assessment involved simulating various precipitation scenarios: drought with less than 66mm annually, normal with 66mm to 110mm, and wet with more than 110mm annually [23]. Additionally, the model was tested across a range of runoff ratios, from 1:10 to 1:30. Toal 5,000 simulation were tested and the resulting data distribution followed a standard bell curve, indicating no abnormalities. Refer to **Fig. K** for wheat and grapevines distribution and **Table D** for crops statistics.

| 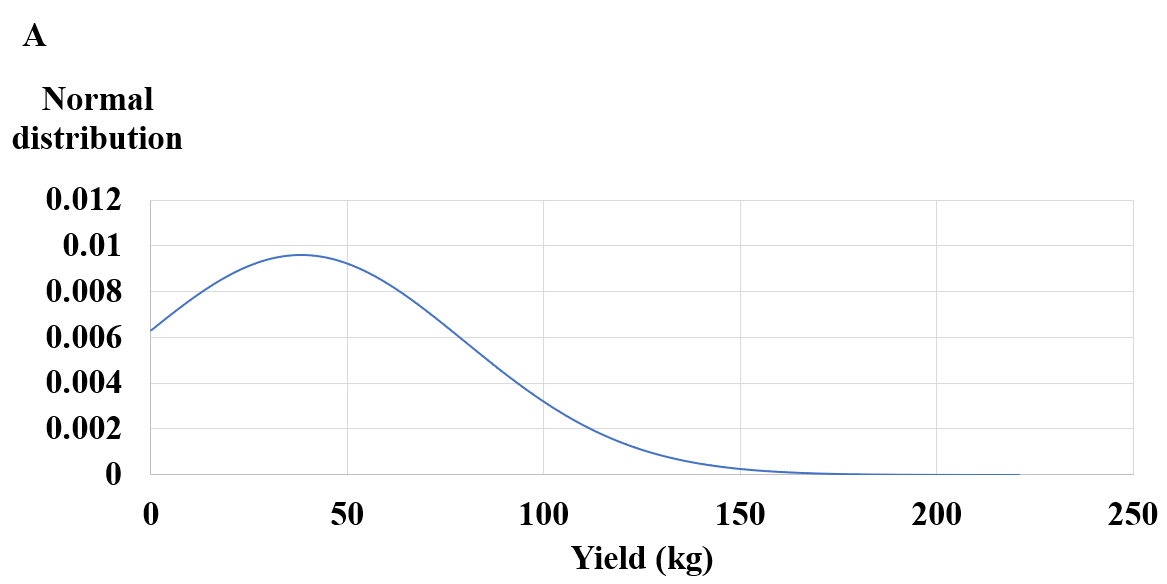 |
| --- |
| 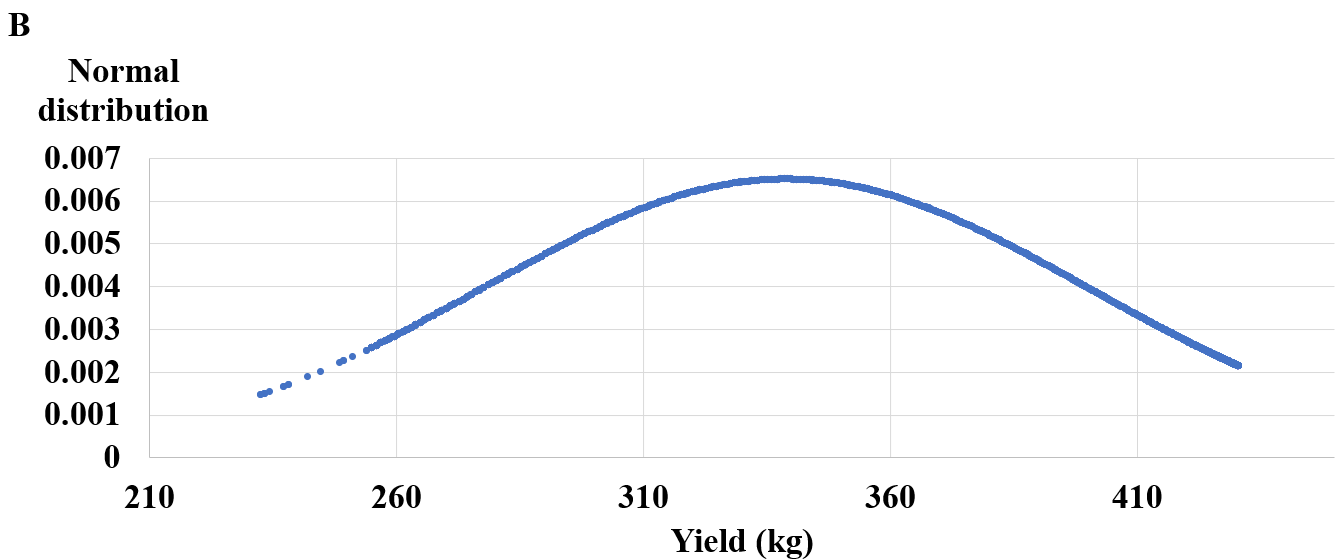 |

**Fig. K : Normal distribution of model crop yield (N=5,000); [A] Wheat, [B] Grapes**

**Table D: AGENTS model crops yield statistics**

| **Crop** | **Mean** | **S.D.** | **Min** | **Max** | **25%** | **50%** | **75%** |
| --- | --- | --- | --- | --- | --- | --- | --- |
| Wheat | 38.3 | 41.7 | 0.1 | 221.0 | 11.1 | 21.4 | 56.3 |
| Grapes | 339.0 | 61.4 | 232.7 | 430.7 | 281.3 | 326.5 | 401.7 |

**S11: AGENTS model BehaviorSpace tests setup**

**Test 1: Yield efficiency as function of runoff ratio**


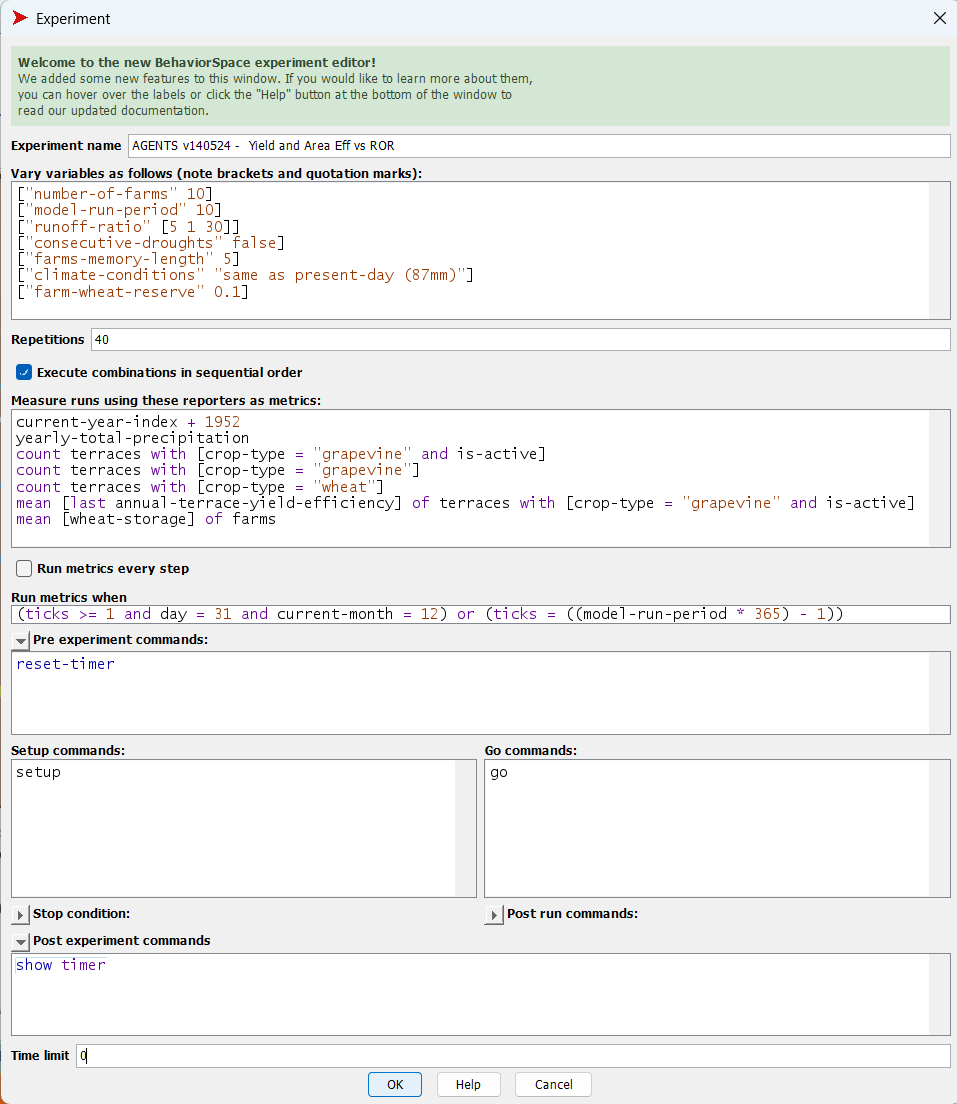


**Fig. L: BehaviorSpace Test 1 configuration (number of simulations 1,040)**

**Test 2: Impact of consecutive droughts (years) on Shivta’s wine industry**


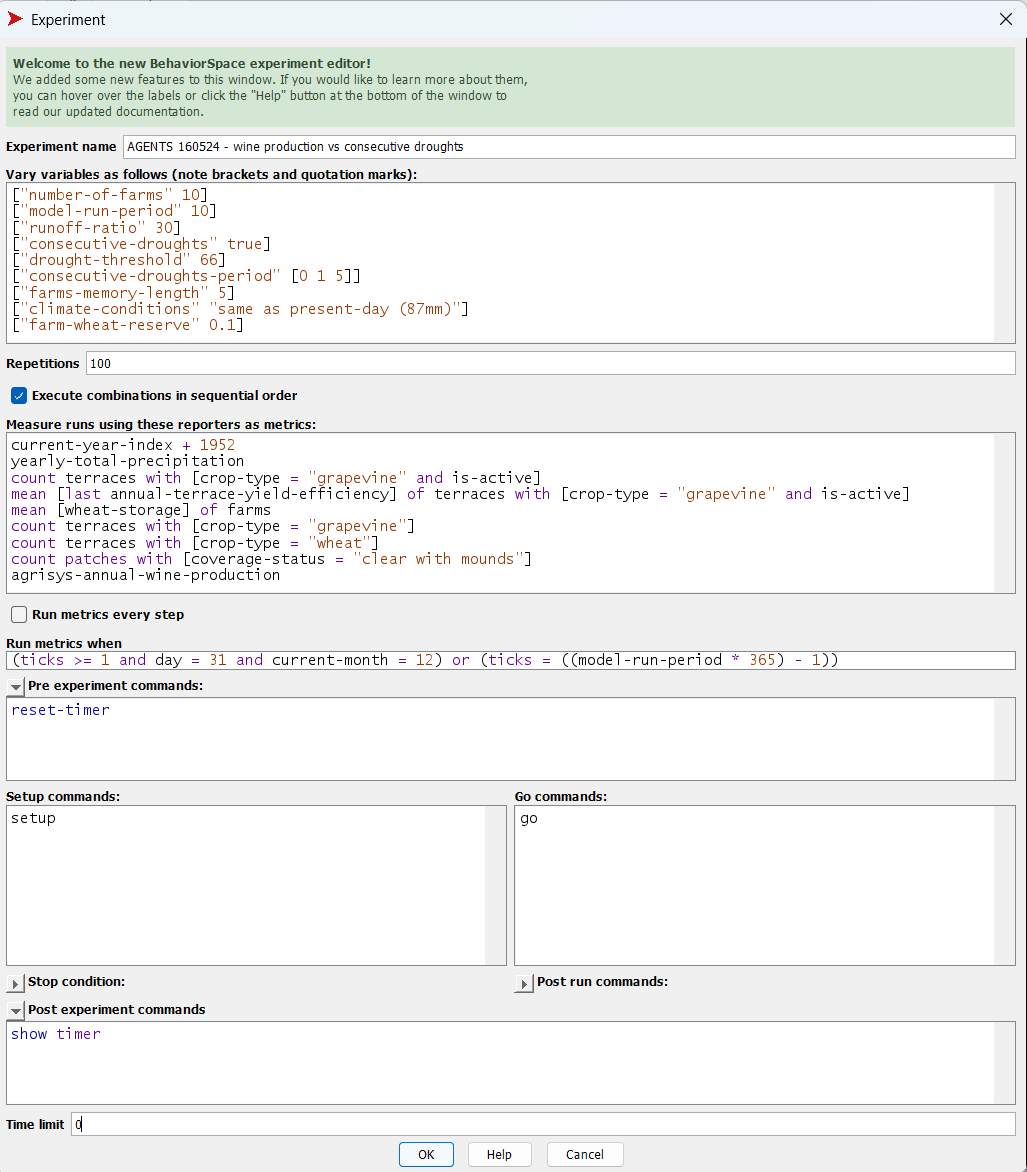


**Fig. M: BehaviorSpace Test 2 configuration (number of simulations 600)**

**Test 3: Shivta’s wine industry recovery time**


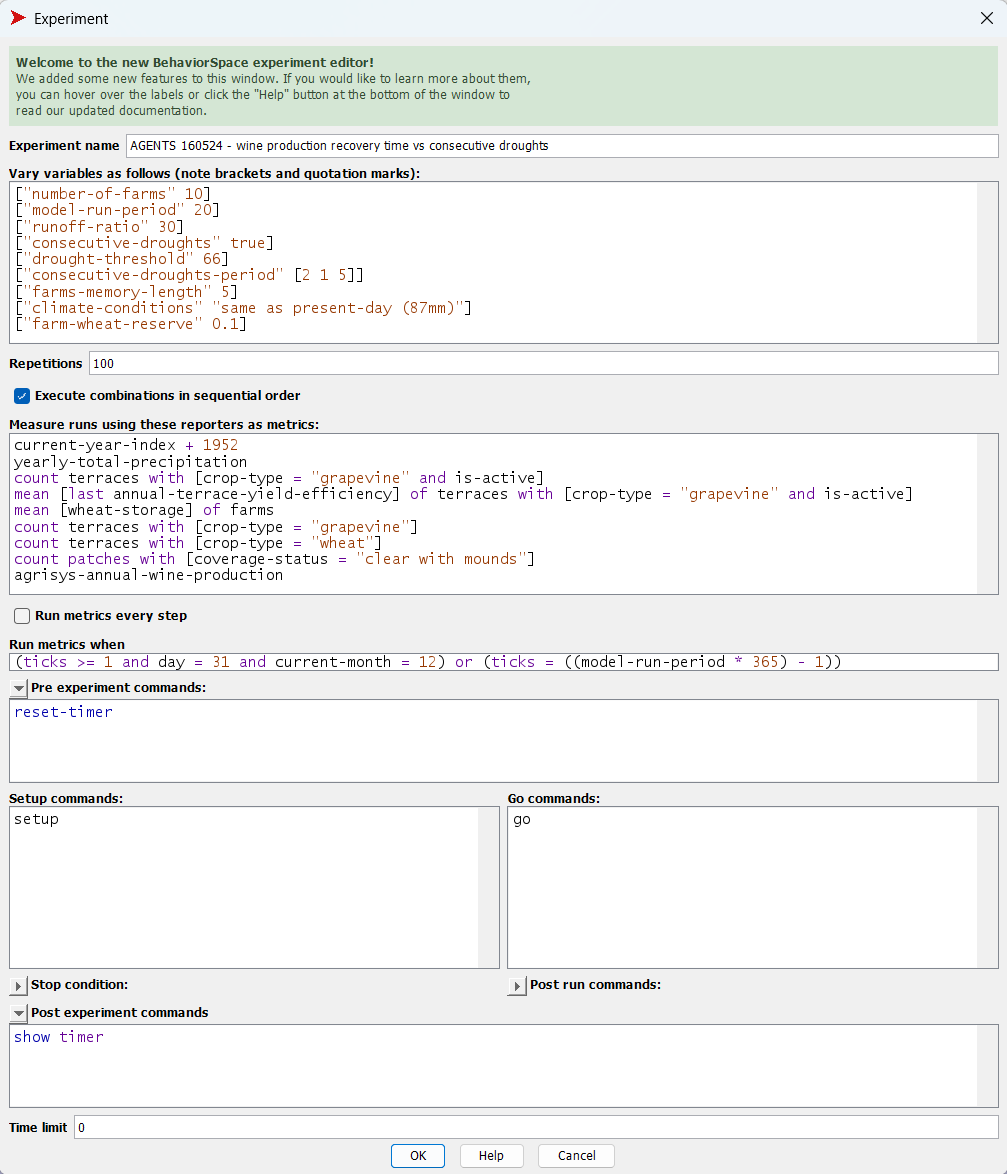


**Fig. N: BehaviorSpace Test 3 configuration (number of simulations 400)**

In this specific test, to ensure that the first 5 years used as the simulation wine production baseline are free of dry years, and that the drought period always begins after the 5th year (in a 20-year simulation), the AGENTS model code was modified. The set-climate-random-years method was adjusted so that the local variable insert-point is set to 5, instead of being assigned a random integer between the model run period and the user-defined length of the dry period prior to executing the simulation from the user interface.

**Test 4: Impact of** **wetter climate on grapevine yield efficiency as function of runoff ratio**


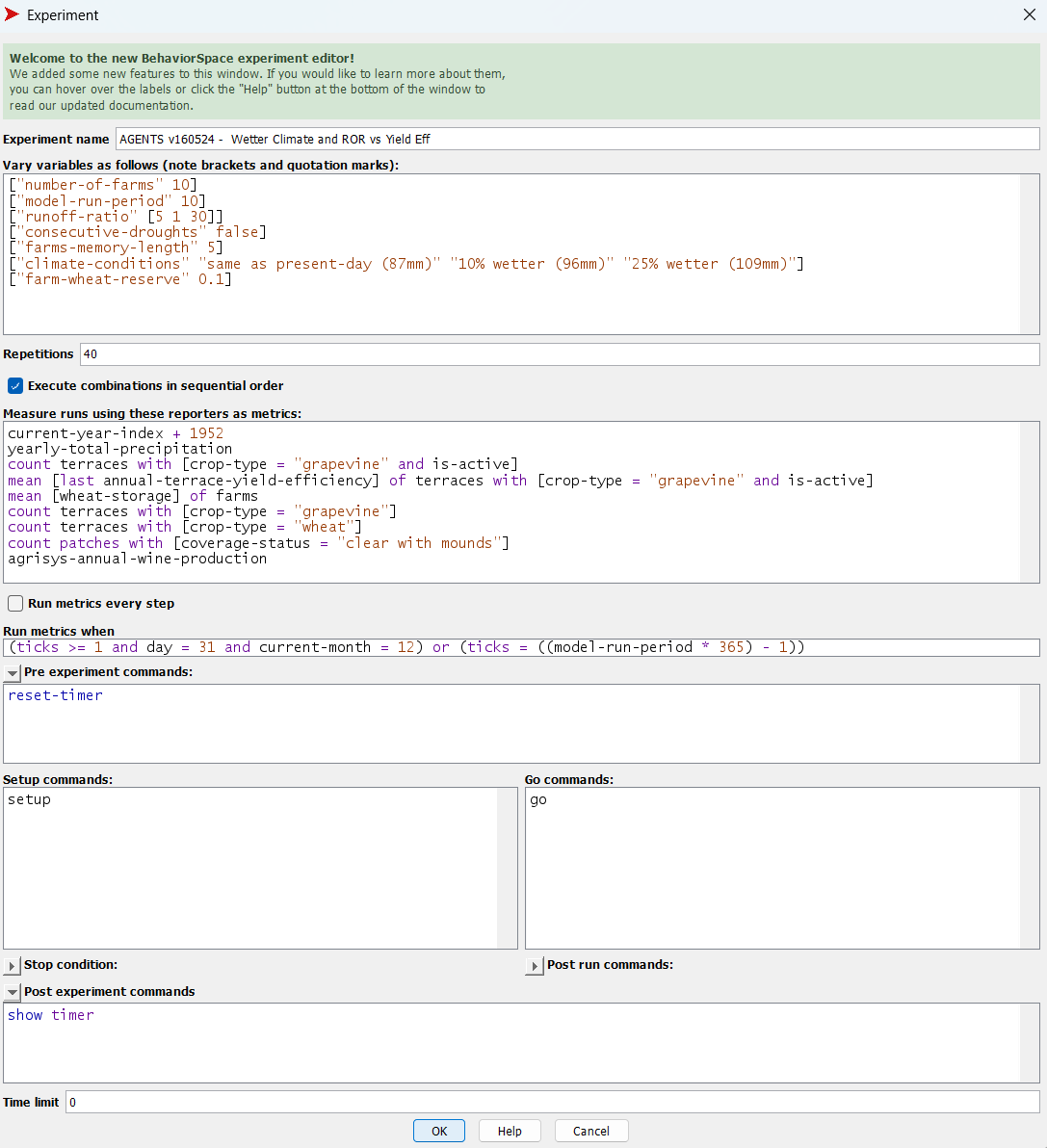


**Fig. O: BehaviorSpace Test 4 configuration (number of simulations 3,120)**

**S12: Runoff Ratio vs. Yield Efficiency** **– Model Setup and SPSS Analysis**

The AGENTS model was configured to assess the impact of the runoff ratio on the yield efficiency of grapevine terraces and the mean annual wine production within the Zetan watershed vineyard system.

The NetLogo BehaviorSpace application was employed to run simulations over random decade-length periods, using precipitation data from the AGENTS model’s precipitation database. Output measurements of annual yield efficiency were recorded. **Table E** provides the setup of the test in the BehaviorSpace application.

**Table E: AGENTS model input and output metrics for testing runoff ratio relationship to grapevine yield efficiency**

| **Inputs** | - Runoff ratio: 5 to 30 with +1 increments - Model run period: 10 years - Consecutive droughts: false - Farms memory length: 5 - Climate conditions: same as present-day (87mm) - Farm wheat reserve: 0.1 |
| --- | --- |
| **Outputs** | - Annual precipitation - Active grapevine terraces - Mean grapevine terraces annual yield efficiency |
| **Metrics taken at:** | End of growing season |
| **Number of scenarios:** | 1,040 random decades (26 runoff-ratio combinations times 40 iterations each ratio value) |

The runoff ratio values were linearly regressed using SPSS against the mean values of annual yield efficiency for grapevine terraces.

A Pearson correlation coefficient was computed to assess the linear relationship between the independent variable, runoff ratio, and the dependent variable, grapevine yield efficiency. The results indicated a significant, strong, positive correlation, r(1) = 0.921, p < 0.001, supporting the initial assumption. To further validate these findings, a simple linear regression was conducted to test if runoff ratio could predict grapevine yield efficiency, as detailed in **Table F**.

**Table F: Linear regression between runoff ratio (independent variable) and grapevine yield efficiency (dependent variables)**

| **Predictors** | **β(t)** |
| --- | --- |
| Grapevine Yield Efficiency | 0.004 (11.603) |
| **R^2^** | 0.849 |
| **Adj. R^2^** | 0.842 |
| **F** | 134.619^**^ |

**p<0.01

The fitted regression model is:

Grapevine Yield Efficiency = 0.2373+ 0.0045 * (Runoff Ratio). The overall regression was statistically significant (R^2^ = 0.849, F(1,24) = 134.619, p < 0.01).

**S13: Drought Period vs. Annual Wine Production – Model Setup and SPSS Analysis**

The AGENTS model was configured to assess the impact of varying lengths of consecutive droughts on overall wine production, with a 10-year no-drought period serving as the baseline.

The NetLogo BehaviorSpace application was used to run simulations over random decade-length periods, utilizing precipitation data from the AGENTS model’s precipitation database. **Table G** provides the setup of the test in the BehaviorSpace application.

**Table G: AGENTS model input and output metrics for testing drought length (years) relationship to annual wine production (liters)**

| **Inputs** | - Number of farms: 10 - Runoff ratio: 30 - Model run period: 10 years - Consecutive droughts: true - Drought threshold: 66mm - Consecutive droughts period: 0, 1 ,2 ,3, 4, 5 (years) - Farms memory length: 5 - Climate conditions: same as present-day (87mm) - Farm wheat reserve: 0.1 |
| --- | --- |
| **Outputs** | - Annual precipitation - Total grapevine terraces - Mean grapevine terraces annual yield efficiency - Mean wheat-storage of farms - Annual wine production |
| **Metrics taken at:** | End of growing season |
| **Number of scenarios:** | 600 random decades (6 drought periods combinations times 100 iterations each drought periods value) |

To enhance differences between the tested drought lengths, the drought lengths were reclassified (at the analysis step; SPSS) into groups: None (0 years), Short (1−2 years), Medium (3−4 years), and Long (5 years).

A one-way ANOVA was performed to compare the effect of the different droughts length groups (None, Short, Medium and Long) on overall Zetan potential wine production. The one-way ANOVA found that there was a statistically significant difference in mean wine production between all groups (F(3,596) = 113.893, p<0.001). Tukey’s HSD (honestly significant difference) Test for multiple comparisons found that the value of potential mean wine production was significantly different between all drought periods tested. Refer to **Table H** and **Table I** for SPSS results summary.

**Table H: One-Way ANOVA test results between droughts length and mean wine production (liters)**

|  | **df** | **F** | **Sig.** |
| --- | --- | --- | --- |
| Between Groups | 3 | 113.893 | <.001 |
| Within Groups | 596 |  |  |
| Total | 599 |  |  |

**Table I: Tukey HSD multiple comparisons test between droughts length (years) and mean wine production (liters)**

| **Droughts Length**  **(I)** | **Droughts Length**  **(J)** | **Mean Difference**  **(I-J)** | **Sig.** | **95% Confidence Interval** | |
| --- | --- | --- | --- | --- | --- |
|  |  |  |  | **Lower Bound** | **Upper Bound** |
| None  (0 years) | Short  (1-2 years) | 1217.4^*^ | 0.000 | 825.0 | 1609.9 |
|  | Medium  (3-4 years) | 2239.2^*^ | 0.000 | 1846.8 | 2631.7 |
|  | Long  (5 years) | 2873.3^*^ | 0.000 | 2420.2 | 3326.5 |
| Short  (1-2 years) | Medium  (3-4 years) | 1021.8^*^ | 0.000 | 701.4 | 1342.3 |
|  | Long  (5 years) | 1655.9^*^ | 0.000 | 1263.5 | 2048.3 |
| Medium  (3-4 years) | Long  (5 years) | 634.1**^*^** | 0.000 | 241.6 | 1026.5 |

* p<0.05

**S14:**  **Drought Period vs. Wine Industry Recovery Time – Model Setup and SPSS Analysis**

The AGENTS model was configured to assess the impact of varying lengths of consecutive droughts on Shivta’s wine industry and its ability to regain pre-drought production levels.

The AGENTS model was run for 20-year simulations, with drought periods of 2, 3, 4, or 5 years (defined as years with less than 66 mm of total annual rainfall) initiated after an initial 5-year non-drought period, which served as the pre-drought wine production capacity baseline**^[[1]](#footnote-1)^**. The remainder of the simulation period, after the drought, consisted of randomly selected non-dry years from the model's precipitation database until a total of 20 years was completed. Each drought length condition was simulated 100 times, resulting in 400 randomized tests. **Table J** provides the test setup in the BehaviorSpace application.

**Table J: Agents model input and output metrics for testing drought length (years) relationship to regain pre-drought production levels (years)**

| **Inputs** | - Number of farms: 10 - Runoff ratio: 30 - Model run period: 20 years - Consecutive droughts: true - Drought threshold: 66mm - Consecutive droughts period: 2 ,3, 4, 5 (years) - Farms memory length: 5 - Climate conditions: same as present-day (87mm) - Farm wheat reserve: 0.1 |
| --- | --- |
| **Outputs** | - Annual precipitation - Total grapevine terraces - Mean grapevine terraces annual yield efficiency - Mean wheat-storage of farms - Annual wine production |
| **Metrics taken at:** | End of growing season |
| **Number of scenarios:** | 400 random 20-years period simulations (4 drought periods combinations times 100 iterations each drought periods value) |

To enhance differences between the tested drought lengths, the drought lengths were reclassified (at the analysis step; SPSS) into groups: Short (2 years), Medium (3−4 years), and Long (5 years). Each drought category (period length) was simulated 100 times of randomly picked 20-years period by the model.

For each simulation, the average wine production over the initial 5-year non-drought period was calculated, reflecting the pre-drought baseline production capacity. This baseline served as a comparison point to assess how quickly the system recovered after the drought.

Next, the wine production for the first year following the drought period (simulations for 2, 3, 4, or 5-year droughts were run 100 times each) was compared with the pre-drought baseline value. If the wine production in that first post-drought year was equal to or higher than the baseline, the time to regain productivity was recorded as 1 year. If it was lower, the average of the first and second post-drought years was calculated. If this average was equal to or higher than the baseline, the recovery time was noted as 2 years. If not, a running average was calculated by adding each subsequent post-drought year’s production to the average until it equalled or surpassed the baseline, at which point the recovery time was recorded as the number of years post-drought.

In addition, for each drought length period (short, medium, and long), all scenarios where 10 years or more were required to regain productivity were counted and divided by the total number of simulations for that period. This was used to establish the probability of failing to regain pre-drought productivity levels even a decade after the end of the drought period.

A one-way ANOVA was performed to compare the effect of drought length on the average time (years) it takes to Shivta’s agriculture system wine production to regain its productivity pre-drought period. The test revealed that there was a statistically significant difference in regaining productivity period between the tested drought length periods factor (F(2,397)=16.510, p < 0.001). Refer to **Table K** for one-way ANOVA test results.

**Table K: One-Way ANOVA test results between regain productivity period (years) and drought length**

| Regain Productivity Period (years) | **df** | **F** | **Sig.** |
| --- | --- | --- | --- |
| Between Groups | 2 | 16.510 | < 0.001 |
| Within Groups | 397 |  |  |
| Total | 399 |  |  |

Tukey’s HSD Test for multiple comparisons found that the value of Regain Productivity Period (years) was significantly different for all different drought periods tested. Refer to **Table L**.

**Table L: Tukey HSD multiple comparisons test between regain productivity period (years) and drought length**

| **Droughts Length**  **(I)** | **Droughts Length**  **(J)** | **Mean Difference**  **(I-J)** | **Sig.** | **95% Confidence Interval** | |
| --- | --- | --- | --- | --- | --- |
|  |  |  |  | **Lower Bound** | **Upper Bound** |
| Short  (2 years) | Medium  (3-4 years) | 0.15 | .006 | -2.678 | -.372 |
|  | Long  (5 years) | -0.66 | .001 | -4.582 | -1.918 |
| Medium  (3-4 years) | Long  (5 years) | -0.82 | .001 | -2.878 | -.572 |

* p<0.05

Additionally, the probability to fail to regain wine production, after over a decade or more from the end of the drought period, was calculated for each drought length scenario.

**S15: Wetter Climate vs. Yield Efficiency – Model Setup and SPSS Analysis**

To test the hypothesis that the Byzantine Negev experienced a wetter climate, enabling the thriving grapevine agriculture and wine production of that era, the model was evaluated under two additional climate scenarios with increased annual precipitation. One scenario featured a 10% increase in rainfall, and the other a 25% increase, over random 10-year periods with varied runoff ratios from 5 to 30 **^[[2]](#footnote-2)^**. A scenario of normal conditions – no manipulation of precipitation model database was made - was used as baseline. **Table M** lists the BehaviorSpace parameters setup (inputs) and metrics taken (outputs) for this test.

**Table M: AGENTS model input and output metrics for testing wetter climates and runoff ratio relationship to grapevine yield efficiency**

| **Inputs** | - Runoff ratio: 5 to 30 with +1 increments - Number of farms: 10 - Model run period: 10 years - Consecutive droughts: false - Farms memory length: 5 - Climate conditions: “same as present-day (87mm)”, "10% wetter (96mm)", "25% wetter (109mm)" - Farm wheat reserve: 0.1 |
| --- | --- |
| **Outputs** | - Annual precipitation - Total grapevine terraces - Mean grapevine terraces annual yield efficiency - Mean wheat-storage of farms - Annual wine production |
| **Metrics taken at:** | End of growing season |
| **Number of scenarios:** | 3120 random decades (3 climate scenarios and 26 runoff-ratio combinations times 40 iterations each climate scenario and runoff-ratio value) |

A one-way ANOVA was performed to compare the effect of wetter climates (precipitation-wise) on the mean yield efficiency of grapevine terraces. The test revealed that there was a statistically significant difference in mean yield efficiency between at least two climate groups factor (F(2,3117)=132.526, p < 0.001). Refer to **Table N** for one-way ANOVA test results.

**Table N: One-Way ANOVA Test results between climate categories and grapevine yield efficiency**

| Grapevine Yield Efficiency | **df** | **F** | **Sig.** |
| --- | --- | --- | --- |
| Between Groups | 2 | 132.526 | 0.000 |
| Within Groups | 3117 |  |  |
| Total | 3119 |  |  |

Tukey’s HSD Test for multiple comparisons found that the value of Grapevine Yield Efficiency was significantly different for all climate groups. Refer to **Table O**.

**Table O: Tukey HSD multiple comparisons test between climate categories and grapevine yield efficiency**

| **Climate Group (I)** | **Climate Group (J)** | **Mean Difference**  **(I-J)** | **Sig.** | **95% Confidence Interval** | |
| --- | --- | --- | --- | --- | --- |
|  |  |  |  | **Lower Bound** | **Upper Bound** |
| same as  present-day | +10% precipitation | -0.029**^*^** | 0.000 | -0.037 | -0.021 |
|  | +25% precipitation | -0.057**^*^** | 0.000 | -0.065 | -0.049 |
| +10% precipitation | +25% precipitation | -0.028**^*^** | 0.000 | -0.036 | -0.019 |

* p<0.05


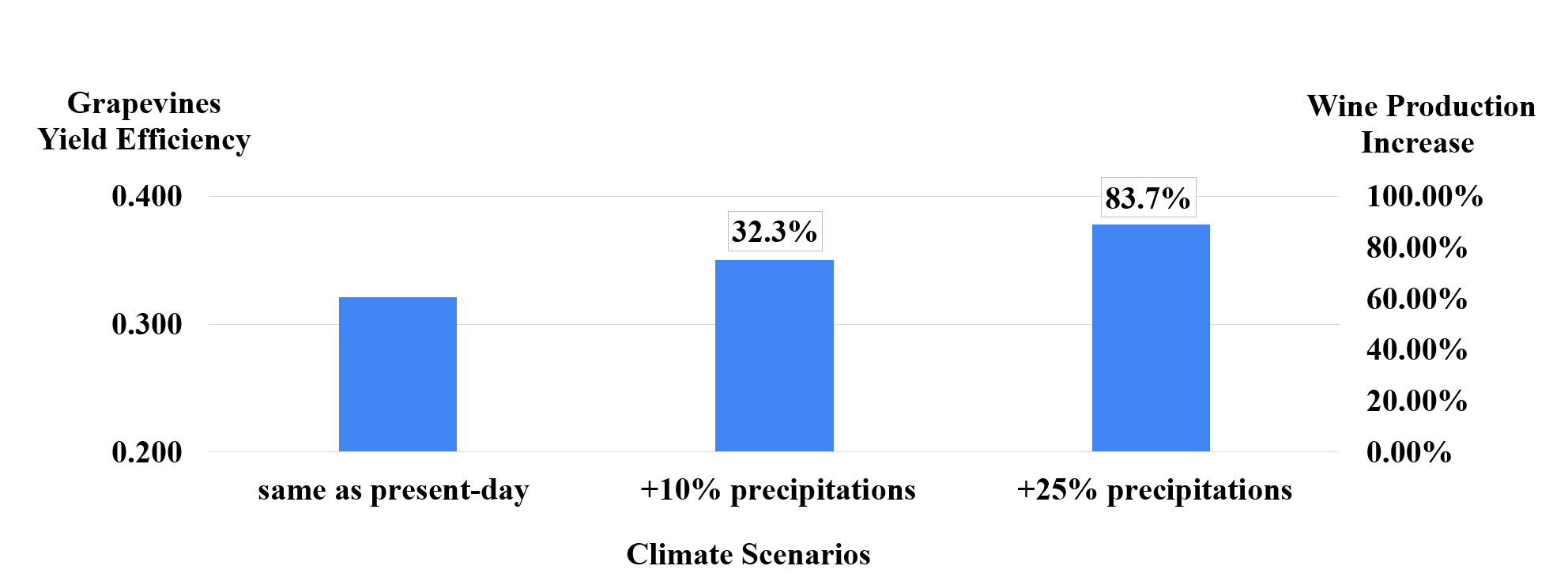


**Fig. P: Grapevine yield efficiency and wine production increase (%) across different climate scenarios**

The blue bars represent grapevine yield efficiency under three climate scenarios: present-day precipitation, 10% increased precipitation, and 25% increased precipitation. The percentage values above the bars indicate the increase in wine production compared to the present-day scenario. For example, under the same-as-present-day precipitation scenario, the yield per 0.1 ha is 154 kg (0.321 yield efficiency × 480 kg/0.1 ha). A 10% increase in precipitation boosts the yield by more than 30%, reaching approximately 200 kg/0.1 ha, while a 25% increase raises it further to around 280 kg/0.1 ha.

Maps throughout this article were created using ArcMap® (version 10.8.2) software by Esri, incorporating the World Imagery basemap [24].

**References**

1. Israel Meteorological Service (IMS). Historical daily precipitation and evaporation data for various stations (1952–2022) [Internet]. Beit Dagan: Israel Meteorological Service; [cited 2023 Nov 22]. Available from: <https://ims.gov.il/en/data_gov>
2. Woodward DE, Hawkins RH, Jiang R, Hjelmfelt, Jr AT, Van Mullem JA, Quan QD. Runoff curve number method: Examination of the initial abstraction ratio. In: World water and environmental resources congress. Philadelphia. 2003pp. 1-16. doi:10.1061/40685(2003)308.
3. Baltas EA, Dervos NA, Mimikou MA. Determination of the SCS initial abstraction ratio in an experimental watershed in Greece. Hydrol Earth Syst Sci. 2007; 11(6): 1825-1829. doi:10.5194/hess-11-1825-2007.
4. Hawkins, R.H., Ward, T.J., Woodward, D.E. and Van Mullem, J.A editors.. Curve number hydrology: State of the practice. American Society of Civil Engineers. Reston VA: ASCE Publications; 2008.
5. Hawkins RH, Hjelmfelt Jr AT, Zevenbergen AW. Runoff probability, storm depth, and curve numbers. J Irrigation Drainage Engineering. 1985; 111(4): 330-340. doi:10.1061/(ASCE)0733-9437(1985)111:4(330).
6. Ajmal M, Waseem M, Kim D, Kim TW. A pragmatic slope-adjusted curve number model to reduce uncertainty in predicting flood runoff from steep watersheds. Water. 2020; 12(5): 1469. doi:10.3390/w12051469.
7. Mockus, V. (1964). National engineering handbook: Section 4 – Hydrology. Washingron DC: United States Department of Agriculture, Soil Conservation Service.
8. Hawkins RH, Moglen GE, Ward TJ, Woodward DE, editors. Updating the curve number: Task group report. In: proceedings of watershed management conference. 2020. Reston, VA: American Society of Civil Engineers.pp. 131-140. doi:10.1061/9780784483060.012.
9. Mishra S, Singh VP. Soil Conservation Service Curve Number (SCS-CN) methodology. In: Singh VP, editor. Water Science and Technology Library. Vol. 42. Dordrecht: Springer; 2003. p. 84-146. doi:10.1007/978-94-017-0147-1_2.
10. Israel Ministry of Agriculture and Rural Development. Soil groups of Israel [Internet]. Jerusalem: Israel Ministry of Agriculture and Rural Development; 2021 May 6 [cited 2023 Nov 22]. Available from: <https://data.gov.il/dataset/soil_groups>
11. Hydrological Service of Israel, Water Authority. Daily average discharge and volumes for all Israel hydrometric stations [Internet]. Jerusalem: Water Authority; [cited 2024 Feb 15]. Available from: <https://data.gov.il/dataset/level_discharge>
12. Unión de Uniones de Castilla-La Mancha. Vendimia: los controles en Castilla-La Mancha se centran en viñas con más de 18.000 y 20.000 Kg/Ha para tintas y blancas, respectivamente [Internet]. Unión de Uniones de Castilla-La Mancha; 2020 Aug 24 [cited 2024 Jul 27]. Spanish. Available from: <https://unionclm.org/2020/08/24/vendimia-los-controles-en-castilla-la-mancha-se-centran-en-vinas-con-mas-de-18-000-y-20-000-kg-ha-para-tintas-y-blancas-respectivamente/>
13. Romero Fresneda R, Moreno García JV, Martínez Núñez L, Huarte Ituláin MT, Rodríguez Ballesteros C, Botey Fullat MR. Comportamiento de las precipitaciones en España y periodos de sequía (periodo 1961-2018) [Internet]. Madrid: Agencia Estatal de Meteorología (AEMET); 2020. Spanish. doi:10.31978/666-20-006-0.
14. Jackson DI, Schuster DF. The production of grapes and wine in cool climates. San Francisco (CA): Board and Bench Pub; 1988.
15. Kedar Y. Ancient Agriculture in the Negev Highlands. Jerusalem: Mosad Bialik; 1967. Hebrew.
16. Lomas J. Efficient utilization of climate resources for rainfed agriculture in Israel. Bull de la Société Botanique de France. 1984; 131(2-4): 501-515. doi:10.1080/01811789.1984.10826690.
17. PlotDigitizer [Internet]. Version 2024 [cited 2024 Jan 23]. Available from: <https://plotdigitizer.com/app>
18. Evenari M, Shanan L, Tadmor N. The Negev: The challenge of a desert. Cambridge: Harvard University Press; 1982.
19. Erasmus CJ. Monument building: Some field experiments. Southwestern J Anthropol. 1965; 21(4): 277-301. doi:10.1086/soutjanth.21.4.3629433.
20. Duncan-Jones R. Economy of the Roman Empire. Cambridge: Cambridge University Presss; 1974.
21. Spurr MS. Arable cultivation in Roman Italy: c. 200 B.C.-c. A.D. 100. London: Society for the Promotion of Roman Studies; 1986.
22. Rosenstein NS. Rome at war: Farms, families, and death in the Middle Republic. Chapel Hill (NC): University of North Carolina Press; 2004.
23. Ran N. Drought in Israel. In: Inbar M, Porat R, editors. Natural Disasters in Israel. Haifa: Department of Geography and Environmental Studies, University of Haifa; 2007. pp. 98-106. Hebrew.
24. Esri. "World Imagery" [basemap]. Scale not given. "World Imagery Map." 2009. Available from:

<https://www.arcgis.com/home/item.html?id=10df2279f9684e4a9f6a7f08febac2a9>

1. To ensure that the drought period always started after 5 years in each simulation, the AGENTS model’s code was modified. Specifically, in the method set-climate-random-years, the line of code that originally read let insert-point random (total-slots - length selected-dry-years) was changed to let insert-point 5. [↑](#footnote-ref-1)
2. In the model, a wetter climate category is produced by multiplying the daily rainfall amounts taken from the precipitation external file by a factor representing the climate category. For example, in the "same as present-day" climate, this factor is 1.0, implying no change to the daily rainfall amounts. For the "10% wetter (96mm)" and "25% wetter (109mm)" climate scenarios, the factors are 1.1 and 1.25, respectively. This adjustment increases the daily rainfall amounts proportionally to simulate wetter climate conditions. [↑](#footnote-ref-2)
