# Supplementary material for: Wine economy in Byzantine Shivta (Negev, Israel): Exploring the role of runoff agriculture and droughts through Agent-Based Modeling
Source: PLoS One. 2025 Jul 10;20(7):e0325204. doi: 10.1371/journal.pone.0325204 (PMC12244680; doi:10.1371/journal.pone.0325204)
Supplement: Attachment — Submitted filename: Response to Reviewers.docx [file pone.0325204.s005.docx]

**Letter to PlosOne editor – response to comments**

Dear editor, please find below our point-by-point response to the concerns and suggestions raised. We provide our responses after each comment in red. We thank the editorial team and all reviewers for the careful reading of the manuscript and the constructive comments provided. The suggestions and clarifications were helpful in improving the accuracy, structure, and contextual framing of the article. Below we outline the changes made in response to each reviewer comment. Where revisions were not made, we provide a brief explanation. We thank you for considering our work for publication in PLoS One. Respectfully yours, Barak Garty.

**Subject:** PLOS ONE Decision: Revision required [PONE-D-25-13772; PONE-D-25-13772R1]

Journal requirements:

When submitting your revision, we need you to address these additional requirements.

1.  Please ensure that your manuscript meets PLOS ONE's style requirements, including those for file naming. The PLOS ONE style templates can be found at

[https://journals.plos.org/plosone/s/file?id=wjVg/PLOSOne_formatting_sample_main_body.pdf](https://track.editorialmanager.com/CL0/https:%2F%2Fjournals.plos.org%2Fplosone%2Fs%2Ffile%3Fid=wjVg%2FPLOSOne_formatting_sample_main_body.pdf/1/010f019616facd39-5b7e2353-7e5b-42a4-8e97-9fc8d934e73b-000000/9g_xGKc2DyQx2cZuPxLnDg1o1mgtpxXKamELDGmBgTA=206)  and

[https://journals.plos.org/plosone/s/file?id=ba62/PLOSOne_formatting_sample_title_authors_affiliations.pdf](https://track.editorialmanager.com/CL0/https:%2F%2Fjournals.plos.org%2Fplosone%2Fs%2Ffile%3Fid=ba62%2FPLOSOne_formatting_sample_title_authors_affiliations.pdf/1/010f019616facd39-5b7e2353-7e5b-42a4-8e97-9fc8d934e73b-000000/Rn91wfer2J-HKV58TXhd6RWiRm8__h2LAgqksozxiac=206)

Response: Done

2. Please note that PLOS ONE has specific guidelines on code sharing for submissions in which author-generated code underpins the findings in the manuscript. In these cases, we expect all author-generated code to be made available without restrictions upon publication of the work. Please review our guidelines at <https://journals.plos.org/plosone/s/materials-and-software-sharing#loc-sharing-code> and ensure that your code is shared in a way that follows best practice and facilitates reproducibility and reuse.

Response: The AGENTS model code and supporting materials (data files and documentation) are publicly available on GitHub:

<https://github.com/BarakGarty/AGENTS>.
The model is also archived with a DOI on COMSES: <https://doi.org/10.25937/b356-wh64>.

3. Thank you for stating the following financial disclosure:

“G. B. O. : European Union European Research Council (ERC) Horizon 2020 Research and Innovation Program (Grant No. 101096539)”

Please state what role the funders took in the study.  If the funders had no role, please state: "The funders had no role in study design, data collection and analysis, decision to publish, or preparation of the manuscript."

If this statement is not correct you must amend it as needed.

Please include this amended Role of Funder statement in your cover letter; we will change the online submission form on your behalf.

Response: "The funders had no role in study design, data collection and analysis, decision to publish, or preparation of the manuscript."

This was also added to revised cover letter that was upload to the revision submission.

4. Thank you for stating the following in the Acknowledgments Section of your manuscript:

“The authors wish to thank Yoav Avni, Yoav Borstein, Hendrick Bruins, Daniel Fuks, Noam Greenberg, Shauly Melitz, Efrat Morin, Moti Zohar for helping us with various aspects of this project and for contributing their knowledge and time. B.G. is grateful to Sir Maurice and Lady Irene Hatter Foundation for providing a research grant. The project received funding from the European Union European Research Council (ERC) Horizon 2020 Research and Innovation Program (Grant No. 101096539).”

We note that you have provided funding information that is currently declared in your Funding Statement. However, funding information should not appear in the Acknowledgments section or other areas of your manuscript. We will only publish funding information present in the Funding Statement section of the online submission form.

Please remove any funding-related text from the manuscript and let us know how you would like to update your Funding Statement. Currently, your Funding Statement reads as follows:

“G. B. O. : European Union European Research Council (ERC) Horizon 2020 Research and Innovation Program (Grant No. 101096539)”

Please include your amended statements within your cover letter; we will change the online submission form on your behalf.

Response: Funding-related text was removed from the Acknowledgments section. All funding information now appears solely in the Funding Statement section, as per journal policy. In addition, our current Funding Statement “G. B. O.: European Union European Research Council (ERC) Horizon 2020 Research and Innovation Program (Grant No. 101096539)”

is sufficient.

5. When completing the data availability statement of the submission form, you indicated that you will make your data available on acceptance. We strongly recommend all authors decide on a data sharing plan before acceptance, as the process can be lengthy and hold up publication timelines. Please note that, though access restrictions are acceptable now, your entire data will need to be made freely accessible if your manuscript is accepted for publication. This policy applies to all data except where public deposition would breach compliance with the protocol approved by your research ethics board. If you are unable to adhere to our open data policy, please kindly revise your statement to explain your reasoning and we will seek the editor's input on an exemption. Please be assured that, once you have provided your new statement, the assessment of your exemption will not hold up the peer review process.

Response: All data used in the research is now publicly available in the GitHub repository: <https://github.com/BarakGarty/AGENTS>. This includes a compressed file titled “Research results.rar,” which contains the Excel datasets derived from the AGENTS model output and used for the statistical analysis performed in SPSS.

6. We note that Figures 1, 2 and Fig. E in your submission contain [map/satellite] images which may be copyrighted. All PLOS content is published under the Creative Commons Attribution License (CC BY 4.0), which means that the manuscript, images, and Supporting Information files will be freely available online, and any third party is permitted to access, download, copy, distribute, and use these materials in any way, even commercially, with proper attribution. For these reasons, we cannot publish previously copyrighted maps or satellite images created using proprietary data, such as Google software (Google Maps, Street View, and Earth). For more information, see our copyright guidelines: <http://journals.plos.org/plosone/s/licenses-and-copyright>.

We require you to either (1) present written permission from the copyright holder to publish these figures specifically under the CC BY 4.0 license, or (2) remove the figures from your submission:

a. You may seek permission from the original copyright holder of Figures 1, 2 and Fig. E to publish the content specifically under the CC BY 4.0 license.

We recommend that you contact the original copyright holder with the Content Permission Form (<http://journals.plos.org/plosone/s/file?id=7c09/content-permission-form.pdf>) and the following text:

“I request permission for the open-access journal PLOS ONE to publish XXX under the Creative Commons Attribution License (CCAL) CC BY 4.0 (<http://creativecommons.org/licenses/by/4.0/>). Please be aware that this license allows unrestricted use and distribution, even commercially, by third parties. Please reply and provide explicit written permission to publish XXX under a CC BY license and complete the attached form.”

Please upload the completed Content Permission Form or other proof of granted permissions as an ""Other"" file with your submission.

In the figure caption of the copyrighted figure, please include the following text: “Reprinted from [ref] under a CC BY license, with permission from [name of publisher], original copyright [original copyright year].”

b. If you are unable to obtain permission from the original copyright holder to publish these figures under the CC BY 4.0 license or if the copyright holder’s requirements are incompatible with the CC BY 4.0 license, please either i) remove the figure or ii) supply a replacement figure that complies with the CC BY 4.0 license. Please check copyright information on all replacement figures and update the figure caption with source information. If applicable, please specify in the figure caption text when a figure is similar but not identical to the original image and is therefore for illustrative purposes only.

The following resources for replacing copyrighted map figures may be helpful:

USGS National Map Viewer (public domain): <http://viewer.nationalmap.gov/viewer/>

The Gateway to Astronaut Photography of Earth (public domain): <http://eol.jsc.nasa.gov/sseop/clickmap/>

Maps at the CIA (public domain): <https://www.cia.gov/library/publications/the-world-factbook/index.html> and <https://www.cia.gov/library/publications/cia-maps-publications/index.html>

NASA Earth Observatory (public domain): <http://earthobservatory.nasa.gov/>

Landsat: <http://landsat.visibleearth.nasa.gov/>

USGS EROS (Earth Resources Observatory and Science (EROS) Center) (public domain): [http://eros.usgs.gov/#](http://eros.usgs.gov/)

Natural Earth (public domain): <http://www.naturalearthdata.com/>

Response: Figures 1, 2 and Fig. E were created using ArcMAP by ESRI with our own layers except the basemap which is Esri ArcMAP software component.

Esri permits the use of its basemaps in publications, including scientific articles, provided proper attribution is included, as outlined in Esri’s guidelines.

In accordance with these requirements, we have taken the following steps:

- Figure Captions: An attribution statement has been added to the captions of all relevant figures: “Basemap source: Esri, Maxar, Earthstar Geographics, and the GIS User Community.”
- Main Text Statement: A general statement was added at the end of the article (after the Supporting Information captions) clarifying that maps throughout the manuscript were created using ArcMap® (version 10.8.2) by Esri, incorporating the World Imagery basemap.
- Reference List Entry: A citation for the basemap was added to the reference list:
  Esri. “World Imagery” [basemap]. Scale not given. “World Imagery Map.” 2009. Available from:

<https://www.arcgis.com/home/item.html?id=10df2279f9684e4a9f6a7f08febac2a9>

We trust that these actions align with both Esri’s attribution policy and PLOS ONE’s CC BY 4.0 licensing requirements.

7. Please review your reference list to ensure that it is complete and correct. If you have cited papers that have been retracted, please include the rationale for doing so in the manuscript text, or remove these references and replace them with relevant current references. Any changes to the reference list should be mentioned in the rebuttal letter that accompanies your revised manuscript. If you need to cite a retracted article, indicate the article’s retracted status in the References list and also include a citation and full reference for the retraction notice.

Response: Reference list was reviewed and all style issues found were corrected.

To academic editor (General changes): In response to reviewer #3's comments regarding the complexity of Table 2, we decided to add a new supplemental file (S8) that explains the values presented in the table, the calculations used to derive them, and the specific input used in the AGENTS model for each agricultural activity. Accordingly, all other supplemental files were renumbered based on the order in which they are cited in the manuscript. Page numbers were also added to the source table, addressing Reviewer #3’s comment regarding precise source referencing.

Reviewer's Responses to Questions

**Comments to the Author**

1. Is the manuscript technically sound, and do the data support the conclusions?
The manuscript must describe a technically sound piece of scientific research with data that supports the conclusions. Experiments must have been conducted rigorously, with appropriate controls, replication, and sample sizes. The conclusions must be drawn appropriately based on the data presented.

Reviewer #1: Yes

Reviewer #2: Yes

Reviewer #3: No

Response: Reviewer 3’s concerns regarding the manuscript’s technical soundness, specifically the cited sources in Table 2 and the calculations used to derive the values in that table, have been addressed. We have thoroughly reviewed Table 2 and sources that did not provide accurate data were removed and we added calculations are now documented in the newly added Supplemental File S8.

2. Has the statistical analysis been performed appropriately and rigorously?

Reviewer #1: Yes

Reviewer #2: Yes

Reviewer #3: No

Response: Reviewer #3 responded “No” to the question regarding whether the statistical analysis was performed appropriately and rigorously, but did not provide specific comments elaborating on this concern. As such, we are not entirely certain what aspect of the analysis was in question.

That said, we revisited the relevant parts of the manuscript to ensure clarity and accuracy. Table 2, which includes key model outputs, was reviewed and revised after identifying some values that were either unclear or based on misleading sources. A new supplemental file (S8) was also added, providing a step-by-step explanation of how each value in Table 2 was calculated.

All model output used in the study is fully available in the GitHub repository, as indicated in the Data Availability section.

3. Have the authors made all data underlying the findings in their manuscript fully available?
The [PLOS Data policy](http://track.editorialmanager.com/CL0/http:%2F%2Fwww.plosone.org%2Fstatic%2Fpolicies.action%23sharing/1/010f019616facd39-5b7e2353-7e5b-42a4-8e97-9fc8d934e73b-000000/6Ksd6P7Z1nG5GI8i-MUMVdJEGW1bEXAvjLmIft1Ldoc=206) requires authors to make all data underlying the findings described in their manuscript fully available without restriction, with rare exception (please refer to the Data Availability Statement in the manuscript PDF file). The data should be provided as part of the manuscript or its supporting information, or deposited to a public repository. For example, in addition to summary statistics, the data points behind means, medians and variance measures should be available. If there are restrictions on publicly sharing data—e.g. participant privacy or use of data from a third party—those must be specified.

Reviewer #1: Yes

Reviewer #2: Yes

Reviewer #3: No

Response: Data is now available on GitHub including the model code and model output used in the SPSS analysis.

4. Is the manuscript presented in an intelligible fashion and written in standard English?
PLOS ONE does not copyedit accepted manuscripts, so the language in submitted articles must be clear, correct, and unambiguous. Any typographical or grammatical errors should be corrected at revision, so please note any specific errors here.

Reviewer #1: Yes

Reviewer #2: Yes

Reviewer #3: Yes

5. Review Comments to the Author

Please use the space provided to explain your answers to the questions above. You may also include additional comments for the author, including concerns about dual publication, research ethics, or publication ethics. (Please upload your review as an attachment if it exceeds 20,000 characters)

Reviewer #1: 1) Page 3: “the thriving wine industry that relied on a strong export market, which began in the late 4th to early 5th centuries and peaked in the 6th century before sharply declining in the mid-7th century CE [22−23]. This decline corresponds with the periods of prosperity and downturn in Negev viticulture and wineries [14].”
Please note the decline in wine production and export from the Central Negev Highlands begins in the mid-6th century. By the mid-7th century, the export market did not just “decline” but had rather ceased altogether. The sentence on Page 3 is somewhat mitigated by the more correct details at the end of the paper on Page 24. Please make the proper correction on Page 3.

Response: The sentence on page 3 (lines 68−69) was revised to more accurately reflect the historical timeline, stating that the decline began in the mid-6th century CE. References were also update.

2) This reviewer is surprised that the authors made no references to recently published evidence from the nearby site and regional capital of Elusa. In general, there is a lack references to nearly any recently published data from sites other than Shivta itself, e.g., Elusa and Avdat. I suggest that this be rectified.

See: Heinzelmann, Michael – Schöne, Christian A. – Schröder, Arne – Wozniok, Diana –Jordan, Florian – Erickson-Gini, Tali – Kühn, Marlu – Langgut, Dafna – Lehnig, Sina. 2022. Elusa – from Nabatean Trading Post to Late Antique Desert Metropolis. Results of the 2015−2020 Seasons. Archäologischer Anzeiger 2022/1. DOI: <https://doi.org/10.34780/di18-ld82>

Response: In response to this comment, we have added the mentioned above reference (Heinzelmann et al., 2022; ref no. 93), which incorporates recently published data from other Byzantine-period Negev settlements beyond Shivta, including Elusa.

3) This reviewer was further surprised to see no mention of an important, relevant reference from Procopius of Gaza (450-526) concerning the condition of vines at Elusa during his lifetime.
See Mayerson P. 1985. The Wine and Vineyards of Gaza in the Byzantine Period. BASOR 257: 75‒80.

Response: The Mayerson (1985) article is in fact cited in the manuscript as reference [1] and appears in the opening sentence of the introduction. We believe the reviewer may have overlooked its inclusion. Additionally, we added to the introduction’s climate context paragraph (p.5, line 119−122) highlighting a letter by Procopius of Gaza that describes drought and wind erosion near Elusa. This historical example complements the discussion of episodic climatic stressors and supports the manuscript’s environmental framing. The relevant source (Mayerson, 1983) was added to the references list (ref np. 31).

4) The map in Figure 1 is incomplete and other sites, particularly the capital of the region at Elusa, needs to be included. In addition, the site name, Nitzana, needs to be revised to: Nessana (as in the Nessana papyri).

Response: Figure 1 was updated to include the Elusa settlement. Additionally, the site label “Nitzana” was corrected to “Nessana” in accordance with the correct historical naming.

5) The map in Figure 2 includes the name: Shivta city. Shivta was not a city but a large village / settlement. Please delete the word city.

Response: The label “Shivta city” in Figure 2 was corrected by removing the word “city.”

6) The same for the table in Figure 3: please delete the word: city.

Response: The text “city” in Figure 3 was removed

7) The resolution of Figure 4 is very poor. This reviewer was unable to read the key.

Response: Figure 4 was updated with enhanced resolution, and the figure legend was enlarged to improve readability and address the reviewer’s concern.

8) The name Zetan also appears as Zethan in Garty’s supplemental file. This and any other place names need to be spelled the same throughout the article and in the figures and supplementary files.

Response: All instances of “Zethan” in the supplementary file were corrected to “Zetan” to ensure consistent spelling throughout the manuscript and accompanying materials.

Reviewer #2: General Assessment

This study presents a well-structured and methodologically rigorous analysis of viticulture in Byzantine Shivta, integrating archaeological, climatic, and economic data with an innovative agent-based modeling (ABM) approach. The manuscript makes a valuable contribution to our understanding of agricultural resilience in marginal environments during Late Antiquity. The interdisciplinary methodology is particularly commendable, offering a quantitative framework for assessing the sustainability of ancient dryland viticulture.

While the manuscript is of high quality and merits publication, a few areas require minor revisions to enhance clarity, completeness, and methodological robustness. These revisions primarily concern historical context, data interpretation, and presentation of visual materials. Given the significance of the study and its solid foundation, I recommend acceptance with minor revisions.

Strengths of the Manuscript

1. Methodological Innovation: The use of an agent-based model (AGENTS) to simulate viticulture dynamics in the Negev Desert is a novel and commendable approach. The integration of NetLogo for environmental and economic simulations adds depth to the analysis.

2. Thorough Analysis of Runoff Agriculture: The discussion of Byzantine dryland farming techniques, including terraces, runoff harvesting, and water management strategies, is well-supported by archaeological and environmental data.

3. Consideration of Climatic Factors: The study provides a strong quantitative basis for assessing the impact of droughts and climatic variability on agricultural resilience in Shivta.

4. Economic Context: The exploration of viticulture as both an agricultural and economic enterprise is well-articulated, providing insights into the economic drivers behind Byzantine settlement sustainability.

Suggested Revisions

1. Incorporation of Additional Historical Context

- The manuscript does not consider the potential impact of the Plague of Justinian (541–549 CE) on the economic downturn of viticulture in Shivta. While recent studies suggest that the plague’s influence was not as catastrophic as once believed (see EISENBERG and MORDECHAI 2020 The Justinianic Plague and Global Pandemics: The Making of the Plague Concept), it remains an important factor to acknowledge when discussing economic resilience in Late Antiquity. A brief discussion of this context would strengthen the manuscript’s historical grounding.

Response: We appreciate the reviewer’s suggestion to consider the Plague of Justinian. However, we prefer not to address this topic, as the extent of its impact on the Negev remains uncertain, and discussing it is not directly relevant to the focus of this manuscript. We believe the existing historical context provided aligns more closely with the scope of the study.

- The correspondence of Procopius of Caesarea with Jerome references droughts and sandstorms in the fourth century that damaged vineyards and increased salinity in local wells (Mayerson 1983, The City of Elusa in the Literary Sources of the Fourth-Sixth Centuries. IEJ 32, 247-53 [252–3]). Including this historical source would complement the climatic data and reinforce the study’s conclusions on environmental challenges.

Response: A sentence was added to the introduction’s climate context paragraph (p.5 lines 119−122) highlighting a letter by Procopius of Gaza that describes drought and wind erosion near Elusa. This historical example complements the discussion of episodic climatic stressors and supports the manuscript’s environmental framing. The relevant source (Mayerson, 1983) was added to the references list.

- Procopius also remarks on the high value of locally produced wine in Byzantine Shivta, which would further support the economic analysis presented in the manuscript.

Response: We consulted the source cited (Mayerson, 1983) and were unable to identify any explicit statement by Procopius of Gaza concerning the value of local wine production in Shivta or neighboring settlements. In the absence of clear textual support for this specific claim, we chose not to make further changes to the manuscript on this point.

2. Comparison with Modern and Pre-Modern Viticulture

- The manuscript compares Byzantine viticulture in Shivta with 17th-century Italian vineyards and contemporary rainfed viticulture in Spain. While these comparisons provide some useful context, they are problematic due to significant differences in climate, soil composition, available technology, and even grape varieties. Given these variables, the direct application of these case studies to Byzantine Shivta should be reconsidered or qualified with greater nuance.

Response: In response to this comment, we have added a clarifying paragraph at the beginning of the Discussion section (lines 547−553) to acknowledge the key differences between Byzantine Shivta and the comparative cases from modern Spain and 17th-century Italy. This paragraph emphasizes that the comparisons are not intended as direct historical analogies, but rather as illustrative benchmarks to explore general patterns in rainfed viticulture under arid conditions.

3. Role of Pigeon Towers and Soil Fertility

- While the manuscript mentions pigeon towers, it does not discuss their role in soil enrichment through bird droppings, which could have significantly influenced vineyard productivity. Including an analysis of this factor would provide a more comprehensive picture of Byzantine agricultural practices.

Response: A sentence was added at the beginning of Section 4.1 (lines 566−571) to note the role of pigeon manure in enhancing soil fertility and supporting crop yields. Relevant archaeological and agronomic references were also added in the reference list).

4. Bibliographic and Editorial Refinements

- The bibliography, while extensive and well-researched, contains minor inconsistencies and typographical errors. A careful editorial review would enhance the manuscript’s overall professionalism and readability.

Response: Bibliography list was reviewed and minor inconsistencies and typographical errors were corrected.

5. Figures and Data Presentation

- The necessity of Figure 3 should be reconsidered, as it does not appear to be critical to the manuscript’s argument. If it does not provide essential data, its removal could streamline the presentation.

Response: Figure 3 was retained as it visually summarizes the GIS-to-NetLogo workflow, which we believe contributes to the transparency and reproducibility of the model-building process. As the integration of spatial data is central to the AGENTS model, we found it appropriate to retain this figure in the revised manuscript.

Conclusion and Recommendation

The manuscript presents a strong and well-supported analysis of Byzantine viticulture in the Negev Desert. The methodological approach is innovative, and the findings provide valuable insights into ancient agricultural sustainability. While the study is highly commendable, addressing the minor revisions outlined above will further strengthen its contribution.

Final Recommendation: Accept with Minor Revisions.

I appreciate the opportunity to review this manuscript and look forward to seeing the revised version.

Reviewer #3: Comments were attached for the editor, and i hope that these will be shared with the authors.

Wearing my ancient historian hat before I considered the modeling, I approached the primary sources that jumped out at me, especially on page 13 which contains table 2: here, there were two issues. The first was that -- when I checked the text of citations -- Varro or Columella etc. did not say what was claimed. I lay out all these issues in the text shared with editors. The second problem was that -- besides the ancient primary sources -- there were a number of references to modern sources like *Negev Challenge of a Desert* or Duncan-Jones on Roman economy that lacked page numbers, and so I could not check the provided figures.

All of these values were critical for the modeling being used in the article, and thereby its conclusions: with disagreement or a lack of reproducibility, there is no other choice but "revise and resubmit."

I will add, however, that the modeling approach used here looks quite interesting! I have some concerns about how applicable it might be for other regions -- or indeed if the ancient primary sources here really have much validity in the ways that the authors want to use them, especially outside Italy. But it's a valuable attempt.

Response: We thank the reviewer for the close and critical examination of Table 2. We acknowledge that in the original submission, several of the values presented were insufficiently supported by the listed sources. In response, we have carefully reviewed and revised Table 2 to ensure each agricultural activity listed is accurately sourced and that its associated labor cost is properly derived.

To improve transparency and clarity, we also created a new supplemental file (S8), which provides a step-by-step breakdown of how the labor cost values were calculated, including primary source references, area conversions, and where applicable, the rationale for choosing specific values or ranges.

We have attached the revised Table 2 as it now appears in the revised manuscript, along with the new Supplemental File S8 for your review.

**Table 2: Farm labor costs per 0.1 hectares in kg of wheat**

| **Activity ^[a]^** | **Man- days ^[b]^** | **Wheat (kg) ^[b]^** | **Source** |
| --- | --- | --- | --- |
| Building a stone terrace | 100 | 90 | [64: 68] |
| Creating wheat field | 4 | 3.6 | [5: 179−190, 69: 284−285] |
| Clearing a runoff field | 20–30 | 18–27 | [5: 132] |
| Wheat field season agriculture activities **^[c]^** | 8 | 7.2 | Columella (*Rust*. 2.12.1)  [70: 329, 71: 138, 72: 68] |
| Wheat sown | -- | 14.8 | Varro (*Rust*. 1.44.1)  Pliny (*HN* 18.12) |
| Vineyard: soil preparation, digging holes and planting vines | 27.8–46.3**^[d]^** | 25.0–41.6 | [70: 331] |
| Vineyard: age 1–3 maintenance (excluding harvest) **^[e]^** | (11.4–15) X3 | (9.2–12.2) X3 | [70: 331, 71: 136] |
| Vineyard:  age 4+  maintenance (including harvest) | 14.2–17.8 | 12.8–16 | [70: 331, 71: 136] |

a. Each year an additional 10% maintenance costs for the infrastructure are forced on each farm [73].
b. When a range of values was available, we chose the mid-point value of the range given.
c. Wheat growing is done in fallow pattern, alternate years, in the model this was coded as only half a wheat filed output is calculated and thus only half the costs are required.
d. Labor based on modern planting density, following rainfed vineyard standards of 3 m between rows and 1.5 m between vines—yielding 2,220 vines/ha or 222 vines per 0.1 ha—consistent with the model’s yield equation
e. First 3 years of vineyard accounted as single cost

**S8: AGENTS Model Labor Cost Calculations for Grapevine and Wheat Farming Tasks**

Creating Wheat Fields

Based on Evenari’s descriptions of ancient farm reconstructions [5:179–190], we estimated that wheat fields were typically enclosed by low earthen walls, approximately 20–30 cm high and twice as wide, forming a mound- or half-circle-shaped border around the field.

In the AGENTS model, a standard field (patch) measures 30 m × 30 m. To enclose such a field, approximately 120 meters of earthen wall would be needed. Assuming a half-circular mound shape with a cross-sectional radius of 0.25 m, the soil volume required for the enclosure is calculated as:

$$\Pi*\left( 0.25m \right)^{2}* 0.5* 120m=11.8m^{3}$$

According to Erasmus [69: 284–285], a worker can excavate approximately 5 cubic meters of soil per day. Therefore, the labor required to construct such an enclosure for a 0.1-hectare wheat field (adjusted from a single patch of 0.09 ha) is calculated as follows:

$$\frac{11.8\frac{m^{3}}{patch}}{5\frac{m^{3}}{man-days}}*\frac{0.1ha}{0.09\frac{ha}{patch}}\approx2.6man-days$$

To account for the additional labor needed to construct runoff diversion features—such as feeder and sub-channels—as described by Evenari [5:179–190], we added approximately 50% (1.4 man-days) to the estimate.

Thus, the total labor required to prepare a 0.1 ha wheat field was estimated at 4 man-days.

Clearing a runoff field

Based on Evenari’s estimate [5:132], clearing a 1-hectare runoff field required between 200 and 300 man-days of labor. Accordingly, clearing a 0.1-hectare field would require 20–30 man-days. The AGENTS model uses the midpoint of this range—25 man-days per 0.1 ha—equivalent to 22.5 kg of wheat in labor cost per runoff plot, as defined by the model’s runoff ratio setting. For example, if the runoff ratio is set to 1:20, the cost of clearing runoff area for each vineyard plot would be 20 times the base cost of 22.5 kg of wheat, totalling 450 kg.

Wheat field season agriculture activities

Columella (*Rust*. 2.12.1) lists several tasks required annually for maintaining one *iugerum* of wheat, totaling 10.5 man-days of labor.

Duncan [70: 329] adds 2–3 man-days per *iugerum* for threshing—a crucial process for separating wheat grains from the chaff—and an additional 1 man-day for cutting straw. This results in a combined total of 3–4 additional man-days.

Spurr [71: 138] and Rosenstein [72: 68] argue that 4–6 more man-days should be added for tasks not explicitly mentioned by Columella, such as manuring, transporting the grain to the threshing floor, gathering hay, and similar duties.

Altogether, these sources suggest a total labor requirement of approximately 20 man-days per *iugerum*, which translates to 8 man-days per 0.1 hectare (noting that 1 *iugerum* ≈ 0.25 hectares).

For wheat farming, the AGENTS model applies a reduced cost in kg of wheat, since fields are managed with a fallow system. Each patch alternates annually between cultivation and rest, meaning only half a patch is active in any given season. Therefore, the cost per 0.1 ha is halved, amounting to 3.6 kg of wheat.

Wheat sown

According to Varro (*Rust*. 1.44.1), wheat was typically sown at a rate of 5 *modii* per *iugerum*. Pliny the Elder (*HN* 18.12) provides a range of wheat varieties from different regions with varying weights per *modius*. We adopted the average value from his accounts—22.5 pounds per *modius*. Using a conversion of 328.9 grams per pound, the total seed weight per *iugerum* can be calculated. To express this in terms of 0.1 hectares (noting that 1 *iugerum* ≈ 0.25 hectares), the equation is as follows:

$$\frac{5\frac{modii}{iugerum}*22.5\frac{pound}{modius}*328.9\frac{gram}{pound}}{2.5\frac{0.1ha}{iugerum}*1,000\frac{gram}{kg}})=14.8\frac{kg wheat}{0.1ha}$$

Thus, sowing one 0.1 ha wheat field would require approximately 14.8 kg of wheat; however, under the fallow system used in the AGENTS model, this amounts to 7.4 kg per 0.1 ha.

Vineyard: soil preparation, digging holes and planting vines

According to Duncan-Jones [70: 331] accounting for Columella work from both *De Re Rustica* and *De Arboribus*, the number of vine holes that can be dug in one man-day per *iugerum* depends on hole size: 12 or14 holes of 4 square feet, 18 holes of 3 square feet, or 20 holes of 2 square feet. This provides a range of 12 to 20 holes per man-day per *iugerum*. Converting this to a 0.1 ha area (noting that 1 *iugerum* ≈ 0.25 ha) yields:

$$Min: 12\frac{holes}{man-day*iugerum}*0.4\frac{iugerum}{0.1ha}=4.8\frac{holes}{man-day*0.1ha}$$

$$Max: 20\frac{holes}{man-day*iugerum}*0.4\frac{iugerum}{0.1ha}=8.0\frac{holes}{man-day*0.1ha}$$

Planting density in the model is based on modern vineyard standards to align with the yield equation, which is also drawn from modern rainfed viticulture. Standard spacing is 3 meters between rows and 1.5 meters between vines within rows, resulting in 2,220 vines per hectare, or 222 vines per 0.1 hectare.

Therefore, digging 222 holes for planting in a 0.1 ha vineyard plot would require:

$$Min: \frac{222\frac{holes}{0.1ha}}{8.0\frac{holes}{man-day*0.1ha}}=27.8 man-days$$

$$Max: \frac{222\frac{holes}{0.1ha}}{4.8\frac{holes}{man-day*0.1ha}}=46.3 man-days$$

In the AGENTS model, we adopted the value of 38 man-days for digging 222 holes, approximately the mid-point of that min-max range, or 34.2kg of wheat in labor costs per 0.1ha.

Vineyard: age 1–3 maintenance (excluding harvest)

Duncan-Jones [70: 331], accounting for Columella work from both *De Re Rustica* and *De Arboribus*, lists the annual agriculture activities required for a vineyard—such as soil loosening, hoeing, and pruning—at 23.5 man-days per *iugerum*. Spur [71: 136] adds an additional 12 man-days per *iugerum* to that estimate to account for harvest labor (7 man-days) and other minor tasks. Duncan-Jones [70: 331], citing other primary sources (Cato, Pliny, Saserna and Varro), suggests that total annual upkeep, including harvest, could reach a maximum of 44.5 man-days per *iugerum*.

To isolate only the maintenance (excluding harvest), the labor estimates per *iugerum* are:

**Minimum**: 23.5 + (12 − 7) = 28.5 man-days, or 11.4 man-days per 0.1 ha

**Maximum**: 44.5 − 7 = 37.5 man-days, or 15.0 man-days per 0.1 ha

The AGENTS model utilizes the midpoint of this range—13.2 man-days per 0.1 ha—as the annual agriculture activities labor cost. To account for the first three years of vine growth, this figure is multiplied by three, resulting in a total of 39.6 man-days. In the AGENTS model code, this labor input is represented as 35.6 kg of wheat equivalent.

Vineyard: age 4+ maintenance (including harvest)

By adding the harvest labor cost of 7 man-days per *iugerum* (equivalent to 2.8 man-days per 0.1 ha), the total labor required for the annual agriculture activities of a vineyard ranges between 14.2 and 17.8 man-days per 0.1 ha. In the AGENTS model, we used the midpoint of this range—16 man-days—which corresponds to 14.4 kg of wheat. This value was applied in the model to represent the annual agriculture activities cost of vineyards aged four years or older.
