# Supplementary material for: Wine economy in Byzantine Shivta (Negev, Israel): Exploring the role of runoff agriculture and droughts through Agent-Based Modeling
Source: PLoS One. 2025 Jul 10;20(7):e0325204. doi: 10.1371/journal.pone.0325204 (PMC12244680; doi:10.1371/journal.pone.0325204)
Supplement: Attachment — Submitted filename: Reviewer comments.docx [file pone.0325204.s002.docx]

**Reviewer’s Comments**

1) Page 3: “the thriving wine industry that

relied on a strong export market, which began in the late 4th to early 5th centuries and peaked in the

6th century **before sharply declining in the mid-7th century CE** [22−23]. This decline corresponds

with the periods of prosperity and downturn in Negev viticulture and wineries [14].”

Please note the decline in wine production and export from the Central Negev Highlands begins in the mid-6^th^ century. By the mid-7^th^ century, the export market did not just “decline” but had rather ceased altogether. The sentence on Page 3 is somewhat mitigated by the more correct details at the end of the paper on Page 24. Please make the proper correction on Page 3.

2) This reviewer is surprised that the authors made no references to recently published evidence from the nearby site and regional capital of Elusa. In general, there is a lack references to nearly any recently published data from sites other than Shivta itself, e.g., Elusa and Avdat. I suggest that this be rectified.

See: Heinzelmann, Michael – Schöne, Christian A. – Schröder, Arne – Wozniok, Diana –Jordan, Florian – Erickson-Gini, Tali – Kühn, Marlu – Langgut, Dafna – Lehnig, Sina. 2022. Elusa – from Nabatean Trading Post to Late Antique Desert Metropolis. Results of the 2015−2020 Seasons. *Archäologischer Anzeiger* 2022/1. DOI: <https://doi.org/10.34780/di18-ld82>

3) This reviewer was further surprised to see no mention of an important, relevant reference from Procopius of Gaza (450-526) concerning the condition of vines at Elusa during his lifetime.

See Mayerson P. 1985. The Wine and Vineyards of Gaza in the Byzantine Period. *BASOR* 257: 75‒80.

4) The map in Figure 1 is incomplete and other sites, particularly the capital of the region at Elusa, needs to be included. In addition, the site name, Nitzana, needs to be revised to: Nessana (as in the Nessana papyri).
5) The map in Figure 2 includes the name: Shivta city. Shivta was not a city but a large village / settlement. Please delete the word city.
6) The same for the table in Figure 3: please delete the word: city.
7) The resolution of Figure 4 is very poor. This reviewer was unable to read the key.
8) The name **Zetan** also appears as **Zethan** in Garty’s supplemental file. This and any other place names need to be spelled the same throughout the article and in the figures and supplementary files.
