# Supplementary material for: Wine economy in Byzantine Shivta (Negev, Israel): Exploring the role of runoff agriculture and droughts through Agent-Based Modeling
Source: PLoS One. 2025 Jul 10;20(7):e0325204. doi: 10.1371/journal.pone.0325204 (PMC12244680; doi:10.1371/journal.pone.0325204)
Supplement: Attachment — Submitted filename: Reviewer Comments.docx [file pone.0325204.s003.docx]

This is a "revise and resubmit" pending significant corrections to Table 2 on page 13, where citations of ancient primary sources do not seem to correspond to the mathematical values provided. These values are critical for the modeling at the core of this article.

Reviewer emailed editor 3/31 hoping to receive from the authors page numbers for the other, modern sources cited, namely Evenari, Shanan, and Tadmor's *Negev Challenge of a Desert*, Duncan-Jones' *Economy of the Roman Empire*, Spurr's *Arable Cultivation in Roman Italy*, and Rosenstein's *Rome at War*. Page references might have cleared up apparent discrepancies, in at least a few cases?

See below.

~~~~~~~~~~~~~~~~~~~~~~

Pg 13 contains Table 2, which is a list of man-day labor equivalents and wheat kg equivalents for various activities pertaining to agriculture, from both modern and ancient sources. “Farm labor costs per 0.1 hectares in kg of wheat”

| **Activity** | **Man-Days** | **Wheat (kg)** | **Source** | **Comments** |
| --- | --- | --- | --- | --- |
| Building a stone terrace | 100 | 90 | 63 |  |
| Creating wheat field | 4 | 3.6 | 5, 68  5 Evenari, Shanan, Tadmor Negev Challenge of a Desert [no pp]  68 Erasmus, Monument building: some field experiments. Southwestern Journal of Anthropology 1965. [no pp] |  |
| Clearing a runoff field | 20-30 | 18-27 | 5 Evenari, Shanan, Tadmor Negev Challenge of a Desert [no pp] |  |
| Wheat field season agriculture activities | 8 | 7.2 | **Columella Rust. 2.12.1:** “And now to reckon up the number of days’ labour required to bring to the threshing-floor what we have committed to the earth, four or five modii of common wheat take up four days’ work of the ploughmen, one of the narrower, two of the hoer for the first hoeing and one for the second, one of the weeder, and one and a half of the reaper—a total of ten and one-half days of labour. Five modii of winter wheat require the same number of days.”  **Columella Rust. 11.2.46:** “During these days, if we have been overwhelmed with work, the same tasks must be carried out which should have been done at the end of May; also all fruit-bearing trees, after being dug round, ought to be earthed up, in order that this work may have been finished before the solstice. Moreover, according to local and climatic conditions, the earth is either broken up for the first time or the process is repeated. If the soil is difficult to work, it takes three labourers a day to break it up for the first time, two for the second time, and one for the third time, but two iugera can be harrowed by one labourer in a day; but if the soil is easy to work, a iugerum can be broken up for the first time by two labourers in a day, for the second time by one, while four iugera can be harrowed by one labourer, since broader ridges can be furrowed in soil which has been already worked. From this reckoning we can infer that in the autumn a hundred and fifty modii of wheat can easily be sown with a single yoke of oxen, and a hundred modii of any kind of pulse.”  [69-71]  69: Duncan Jones, Economy of the Roman Empire [no pp]  70: M.S. Spurr, *Arable cultivation in Roman Italy c. 200 B.C.-c. A.D. 100* (London: Society for Promotion of Roman Studies, 1986) [no pp]  71: N.S. Rosenstein, *Rome at war: farms, families, and death in the Middle Republic* (UNC Press, 2004) | Columella 2.12.1 includes a series of tasks for wheat agriculture: ploughing, hoeing, weeding, reaping, to total 10.5 days. And he says that five modii of winter wheat requires the same (per iugerum, as stated elsewhere by Columella e.g. see below 1.44.1).  1 iugerum = 0.25ha  So 10.5 md x 4 = 42 md per hectare.  **Because 0.1ha is the unit of comparison, then 42 / 10 = 4.2 md per 0.1ha. This is half the value provided by authors.**  Columella 11.2.46 gives a different set of rates, specific to breaking up different kinds of soil: these total 6 md per iugerum for difficult soil and 3.25 md per iugerum for easy soils.  Because 1 iugerum = 0.25ha, then  6md difficult soil * 4 = 24md per hectare in difficult soils  3.25 md for easy soils x 4 = 13 md per hectare in easy soils  Because 0.1ha is the unit of comparison, then  **24 / 10 = 2.4md per 0.1ha difficult soils**  **13 / 10 = 1.3md per 0.1ha easy soils**  These figures also do not match with those provided by the authors. Perhaps they added the two sets [e.g. 4.2 + 2.4] bringing us closer, but Columella 2.12.1 explicitly includes ploughing work to break up soils. No surprise too, that he’s inconsistent as ancient authors often are! **In any event, the figures provided by authors do not match up with ancient sources.** |
| Wheat sowing | N/a | 13.4 | **Varro Rust. 1.44.1: “**Beans are sowed 4 modii to the iugerum, wheat 5, barley 6, spelt 10, the amount being a little more or less in some localities; more being sowed on rich ground and less on thin. You should therefore note the amount that is usually sowed in the district and follow this practice; for the locality and the type of soil is so important that the same seed in one district yields tenfold and in another fifteen-fold—as at some places in Etruria.” | What does 13.4kg of wheat here represent? It seems to be the sowing rate (that is, kg wheat sown per 0.1ha): authors might change the label from “wheat sowing” to “wheat sown”  If Varro says 5 modii of wheat per iugerum, and a modius is 8.73 L, then 5 modii x 8.73 L = 43.65 liters.  **Conversion to weight requires a bulk density, which is dependent on moisture and other factors. 34.5kg / 43.65 L = 0.79 kg/liter as bulk density, which seems reasonable though they do not provide a citation for it.** |
| Vineyard: soil preparation, digging holes and planting vines | 36.4-39.6 | 32.8-35.6 | **Columella Rust. 3.13.3-4:** “Those, moreover, whose habit it is to set the vine in planting-holes, after excavating the earth for about three feet in length and two in depth—to as great a width as that of the iron spade permits, lay the shoots on both sides along the walls of the ditches and bend them to stand erect at the opposite ends of the holes; and then, allowing two eyes to project above ground, they replace the earth about it and level off the rest. This they do in the same line, leaving undug skips of the same number of feet, until they come to the end of the row. Then leaving a space, according to each man’s habit of cultivating with either plough or two-pronged mattock, they set the next row. And if the earth is merely turned by a spade-man, the minimum distance between rows is five feet, and seven is the maximum; but if with oxen and plough, the minimum is seven feet, while ten is large enough. Yet some set all their vines at ten-foot intervals, in the form of a quincunx,[^a^](https://www-loebclassics-com.proxy-remote.galib.uga.edu/view/columella-agriculture/1941/pb_LCL361.311.xml#note_LCL361_311_a) so that the ground may be broken up by diagonal and cross ploughing in the manner of fallow land. This sort of vineyard is not to the advantage of the farmer except where, in very fertile soil, the vine is of large growth. But those who dread the expense of trenching the ground, and yet wish to imitate that trenching in some measure, run straight furrows to a width of six feet, leaving alternate strips of equal width; then they dig the furrows and deepen them to three feet, and place the vines or shoots along the sides of the hollows.  **Columella Rust. 4.1.6:** “And on this account, in wedding vines to trees, no one prepares a planting-hole less than three feet deep for the vines. But it is with little insight into the interests of husbandmen, that he remarks that there are peculiar advantages in shallow planting, in that plants grow up quickly when they are not wearied and pressed down by a great weight of soil, and that plants which are lightly supported become more productive. For both these arguments of Julius Atticus are overthrown by the case of planting beside trees, which obviously makes the vine much stronger and more fruitful; which would not be the case if the plants were suffering from being sunk too deep.”  **Columella Rust. 5.3.1-9:** “Having grasped what may be called the first principles of this kind of calculation, we shall have no difficulty about entering upon the measurement of pieces of land, with the various kinds of which it is a long and arduous task to deal at this point. I will now also add, in addition to those which I have already set forth, two rules which husbandmen often employ in the setting out of plants.  Suppose that you have a piece of land 1200 feet long and 120 feet wide, in which vines have to be so arranged that five feet are left between the rows. How many plants, I ask, are necessary when spaces of five feet are required between the plants. I take a fifth of the length, which makes 240, and a fifth of the breadth, which makes 24. To each of these numbers always add one unit, which forms the outermost row, and which they call the angular row; one number, therefore amounts to 241, the other to 25. Multiply these figures as follows: 25 times 241 makes 6025. This, you will say, is the number of plants required.  Similarly, if you wish to set them six feet apart, you will take a sixth of the longitude (which is 1200), that is 200, and a sixth of the breadth (which is 120), that is 20. To each of these figures you will add what I called the angular units. The numbers are 201 and 21. These sums you will multiply together, 21 times 201, and you will get 4221. This, you will say, is the number of plants required.  Similarly, if you wish to set them seven feet apart, you will take a seventh of the length and of the breadth and you will add the angular units, and by the same method and the same arrangement you will make up the number of the plants. In a word, however many feet you have decided for the distance between the plants, you will take the total length and the total breadth and add the units mentioned above. This being so, it follows that the iugerum of land, which is 240 feet long and 120 feet broad, if the distance between the plants is three feet (and this we consider to be the smallest distance which should be left when planting vines), will accommodate 81 plants in its length, and in its breadth, with a distance of five feet between them, it will hold 25 plants. These numbers when multiplied together make 2025.  If the vineyard is arranged with intervals of four feet each way, the row which runs lengthways will contain 61 plants, and the row which runs breadthways 31 plants; this gives 1891 vines to a iugerum. If the vineyard be laid out so that there are intervals of four feet lengthways and five feet breadthways, the row which runs lengthways will have 61 plants and that which runs breadthways 25 plants. If the planting is carried out with intervals of five feet, the row will contain 49 plants lengthways and 25 breadthways; the two numbers multiplied together make 1225. If, however, you have decided to lay out the same area with the vines at intervals of six feet, there is no doubt that 41 vines must be assigned to the length and 21 to the breadth; these numbers multiplied together make a total of 861. But if the vineyard has to be arranged with intervals of seven feet, a row will accommodate 35 heads lengthways and 18 breadthways; these numbers multiplied together make 630, and this, we shall say, is the number of plants which must be got ready. But if the vineyard is to be planted with intervals of eight feet, a row will accommodate 31 plants lengthways and 16 breadthways; these numbers multiplied together make 496. If the interval is to be nine feet, a row9 will hold 27 plants lengthways and 14 breadthways; these numbers multiplied together make 378. With intervals of ten feet, a row will hold 25 plants lengthways and 13 breadthways; these numbers multiplied together make 325. So that our discussion may not be infinitely prolonged, we shall carry out our planting by using the same proportion to suit the wider spacing which any one of us prefers. Let what we have said about the measurement of land and the number of plants suffice. I now return to my proposed order of subjects.”  **Columella Rust. 11.2.17:** “The trenching too,17 which was begun in December or January, must now be finished and the ground so treated planted with vines. A iugerum of land is trenched, in such a way as to be dug to a depth of three feet, by eighty labourers working for one day, or to the depth of two and a half feet by fifty labourers, or to the depth of a double mattock, which is two feet, by forty labourers.”  **Columella Rust. 11.2.28:** “It is the time when planting-holes of every kind will have to be made for the trees which you are going to plant in the autumn; if the ground is suitable, one man in a day can make fourteen of them measuring four feet each way or eighteen measuring three feet each way. But for planting vines or trees of no great growth a furrow a hundred and twenty feet long and two feet broad ought to be sunk to a depth of two feet and a half, and this task a labourer similarly carries out in a day.” | The trenching and planting-hole methods described by these sources are alternative modes of viticulture.  Trenching here is described by Columella 11.2.17: “a iugerum of land is trenched…to a depth of three feet by 80 laborers working for one day”.  1 iugerum = 0.25ha  80 md for 1 iugerum or 0.25 🡪 80 md * 4 for one hectare is 320, and divided by 10 for 0.1ha = **32 mandays for 0.1ha, for trenching alone**.  Digging planting-holes is described by Columella 11.2.28: “one man in a day can make 18 holes measuring 3 feet each way”. Columella 5.3 describes the spacing, of five feet each between holes and rows: one iugerum 240 x 120 feet long could provide space for 1225 vines. [Note that Columella 5.3 also describes a piece of land 1200 x 120 feet long that requires 6025 vine plants; this is rather larger than a iugerum but proportionately it can be worked out that that 1205 plants are needed per iugerum, just slightly smaller than estimate here by spacing method]. Because one iugerum is 0.25 ha and can carry 1225 plants, then 0.1ha can house 490 vines. **Digging 18 holes per day, this is 27.2 man-days per 0.1ha.**  **Columella 5.3 provides for other alternatives in vine spacing: 4x5 (1525 vines per iugerum or 610 vines per 0.1ha = 33.89 man-days per 0.1ha) and 4x4 (1891 vines per iugerum or 756 vines per 0.1ha = 42 man-days per 0.1ha).**  **Whether digging holes (ranging from 27.2 md / 0.1ha with 5x5 spacing, 33.89 md / 0.1ha with 4x5 spacing, or 42 md / 0.1ha with 4x4 spacing) or trenching (32 md / 0.1ha),**  **The range of numbers here from 27.2 – 42 md / 0.1ha embrace but are still rather different than those provided by authors!**  **I won’t even get into the wheat equivalents; I have no idea how those were worked out from the sources here.** |
| Vineyard: age 1-3 maintenance excluding harvest | (11.4 – 15) x 3 | (9.2 – 12.2) x 3 | **Columella Rust. 11.2.44:** From May 15th to June 1st you should again dig over an old-established vineyard before it begins to flower, and trim it and all the other vineyards. If you do this rather frequently, one boy-worker will trim a iugerum of vineyard in a day. In some districts sheep are sheared at this time, and a survey is taken of the stock which has been born and that which has been lost. Also he who sows lupines for manuring the land now finally turns it in with the plough. | This source refers *only* to trimming, not to any other activities required for maintenance (which are described in the rest of Book 11 but are complicated and demand further analysis for adducing labor rates as here)  **With one iugerum being ¼ of a hectare, that’s 4 man-days per hectare. One tenth of a hectare is the unit of comparison here, so 4md per hectare / 10 = 0.4 m-d per 0.1ha. That is vastly less than the 34.2 to 45 man-days per 0.1ha claimed here.**  Perhaps the authors intended for other activities besides trimming, but the citation does not reflect that. |
| Vineyard age 4+ maintenance including harvest | 14.2-17.8 | 12.8-16 | **Cato Agr. 11.1:** “This is the proper equipment for a vineyard of 100 iugera: An overseer, a housekeeper, 10 labourers, 1 teamster, 1 muleteer, 1 willow-worker, 1 swineherd—a total of 16 persons; 2 oxen, 2 draft donkeys, 1 for the mill; 3 complete presses, vats for holding five vintages of 800 cullei…”  **Pliny HN 17.215: “**Props should be placed against a which it may catch hold of and climb up if they are taller than it is. It is said that espaliers for vines of high quality should be cut about March 19th–23rd, and, if it is intended to keep the grapes for raisins, when the moon is on the wane, but that those cut between the old moon and the new are immune from all kinds of insects. Another theory holds the opinion that vines should be pruned by night at full moon when the moon is in the Lion or Scorpion or Archer or Bull; and in general that they should be planted when the moon is at full, or at all events is waxing. **In Italy a gang of ten farmhands is enough for a hundred iugera of vineyard.”**  **Varro Rust. 1.18.2:** “Saserna states that one man is enough for eight iugera, and that he ought to dig over that amount in forty-five days, although he can dig over a single iugerum with four days’ work; but he says that he allows thirteen days extra for such things as illness, bad weather, idleness, and laxness. Neither of these writers has left us a very clearly expressed rule. For if Cato wished to do this, he should have stated it in such a way that we add or subtract from the number proportionately as the farm is larger or smaller.” [note that context here is disagreement with Cato and Saserna… further down at 6 Varro notes that “Wherefore the proper number and variety of slaves must be determined by another method, and Saserna is more to be approved in this matter; he says that each iugerum is enough to furnish four days ‘work for one hand. But if this applied to Saserna’s farm in Gaul, it does not necessarily follow that the same would hold good for a farm in the mountains of Liguria.”]  [69-70]  69: Duncan Jones, *Economy of the Roman Empire* [no pp]  70: M.S. Spurr, *Arable cultivation in Roman Italy c. 200 B.C.-c. A.D. 100* (London: Society for Promotion of Roman Studies, 1986) [no pp] | Cato says 16 workers (presumably year-round) are sufficient for 100 iugera = 25 hectares.  16 workers x 365 days = 5840 mandays per year.  5840 mandays / 25ha = 233.6 man-days per hectare per year.  Because 0.1ha is the unit of comparison, that’s 233.6 man-days per hectare per year = 23.36 mandays per 0.1ha. **This is 31% higher than the authors’ range.**  Pliny says that 10 workers (presumably year-round) are sufficient for 100 iugera of vineland = 25 hectares.  10 workers x 365 days = 3650 mandays per year for 100 iugera or 25 hectares.  3650 mandays / 25 hectares = 146 mandays per hectare.  Because 0.1ha is the unit of comparison, that’s 14.6 md per 0.1ha. **This fits at the very bottom of the authors’ stated range, but it’s unclear where their low or high numbers come from with the primary sources considered!**  **And once again I have no idea how the wheat equivalents were calculated from this data.**  **The modern sources lack page numbers, and cannot be easily vetted.** |

** have emailed editor Joe Uziel 3/31 asking if authors can provide page numbers for their figures in the modern sources.
